# Comparative Efficacy and Safety of Four Different Spontaneous Breathing Trials for Weaning From Mechanical Ventilation: A Systematic Review and Network Meta-Analysis

**DOI:** 10.3389/fmed.2021.731196

**Published:** 2021-11-22

**Authors:** Li-Juan Yi, Xu Tian, Min Chen, Jin-Mei Lei, Na Xiao, Maria F. Jiménez-Herrera

**Affiliations:** ^1^Nursing Department, Hunan Traditional Chinese Medical College, Zhuzhou, China; ^2^Nursing Department, Universitat Rovira i Virgili, Tarragona, Spain

**Keywords:** spontaneous breathing trials, weaning, mechanical ventilation, meta-analysis, systematic review

## Abstract

**Background:** Spontaneous breathing trial (SBT) has been used to predict the optimal time of weaning from ventilator. However, it remains controversial which trial should be preferentially selected. We aimed to compare and rank four common SBT modes including automatic tube compensation (ATC), pressure support ventilation (PSV), continuous positive airway pressure (CPAP), and T-piece among critically ill patients receiving mechanical ventilation (MV).

**Methods:** We searched PubMed, EMBASE, and the Cochrane Central Register of Controlled Trials (CENTRAL) to identify studies that investigated the comparative efficacy and safety of at least two SBT strategies among critically ill patients up to May 17, 2020. We estimated the surface under the cumulative ranking curve (SUCRA) to rank SBT techniques, and determined the certainty of evidence using the Grading of Recommendations Assessment, Development and Evaluation method. Primary outcome was weaning success. Secondary outcomes were reintubation, SBT success, duration of acute care, and intensive care unit (ICU) mortality. Statistical analysis was conducted by using RevMan 5.4, Stata, and R software.

**Results:** We enrolled 24 trials finally. Extubation success rate was significantly higher in ATC than that in T-piece (OR, 0.28; 95% CI, 0.13–0.64) or PSV (OR, 0.53; 95% CI, 0.32–0.88). For SBT success, ATC was better than other SBT techniques, with a pooled OR ranging from 0.17 to 0.42. For reintubation rate, CPAP was worse than T-piece (OR, 2.76; 95% CI, 1.08 to 7.06). No significant difference was detected between SBT modes for the length of stay in ICU or long-term weaning unit (LWU). Similar result was also found for ICU mortality between PSV and T-piece. Majority direct results were confirmed by network meta-analysis. Besides, ATC ranks at the first, first, and fourth place with a SUCRA of 91.7, 99.7, and 39.9%, respectively in increasing weaning success and SBT success and in prolonging ICU or LWU length of stay among four SBT strategies. The confidences in evidences were rated as low for most comparisons.

**Conclusion:** ATC seems to be the optimal choice of predicting successful weaning from ventilator among critically ill patients. However, randomized controlled trials (RCTs) with high quality are needed to further establish these findings.

## Introduction

Successful weaning from mechanical ventilation (MV) refers to the gradual transition from total artificial ventilation support to spontaneous breathing. Delayed disconnection from ventilator can be associated with numerous complications, such as ventilator-associated pneumonia, airway trauma, and multiple-organ failure (MOF) (1–3). The risk of complications and mortality may accrue with increasing duration of MV (4). Therefore, it is essential to timely and safely liberate patients from mechanical ventilator when they have restored the ability of spontaneous breathing (5–8).

Spontaneous breathing trial (SBT) is one of the most common approaches to facilitate the disconnection from MV (9). Evidence-based guidelines have also recommended to conduct SBT immediately before extubation for the purpose of assessing whether a patient is able to restore the ability of spontaneous breath, and thus determine the optimal time for disconnecting from ventilator (10–14). At present, T-piece, continuous positive airway pressure (CPAP), pressure support ventilation (PSV), and automatic tube compensation (ATC) are the most common ventilation techniques (11, 12, 15–20). SBT strategies focused in this study can be categorized into three categories as follows: (a) providing constant or dynamic ventilatory support to counteract the resistance of the endotracheal tube such as PSV and ATC (21–23), (b) providing continuous positive pressure in both inspiration and expiration to enhance breathing mechanics and reduce the effort needed by mechanically ventilated patients with airflow obstruction such as CPAP (24–27), and (c) accelerating spontaneous breath of patients without positive pressure support such as T-piece, which is related to more frequent respiratory activity and consumption of more oxygen (28, 29).

Disconnection from mechanical ventilator should be conducted when patients do not experience any intolerable events after accomplishing SBT (5). However, it is still conflicting as to which SBT should be preferentially selected in route daily practice. Although many studies comparing the efficacy and safety of more than two SBT strategies have been published (21, 22, 30–40, 94), only one (32) investigated the comparative efficacy and safety of all available SBT modes simultaneously at one analysis and suggested that ATC might be superior to T-tube or CPAP for extubation success and tolerance. It must be noted that the reliability of these findings should be interpreted cautiously because these findings were generated from a single-center trial with a limited sample size. Moreover, standard ventilators were utilized in this study, which deeply compromised the accurate compensation of ATC, provided an for the workload imposed by the tube (32). Furthermore, a direct meta-analysis (41) evaluated the efficacy of common types of SBTs, and revealed that patients receiving PSV (vs. T-tube) were more likely to obtain successful extubation. However, this meta-analysis ignored the variations in populations (children and adult) and study design (randomized and quasi-randomized trials) and only provided fragmentary pairwise results, all of which limited the value of pooled results.

As an expansion of direct meta-analysis, network meta-analysis (NMA) can simultaneously combine multiple treatments (more than two) in an individual analysis at one time. Consequently, it can make comprehensive assessments of the differences between all available treatments and clearly display the hierarchies of available treatments (42, 43). We therefore conducted the present NMA of randomized controlled trials (RCTs) to comprehensively compare and rank four common SBT strategies among critically ill patients who required invasive MV for at least 24 h through evaluating weaning success, reintubation, SBT success, duration of acute care, and ICU mortality.

## Methods

We conducted the present study and reported all pooled results according to the preferred reporting items for systematic review and meta-analysis for NMA (PRISMA-NMA) (44). The completed PRISMA-NMA checklist is available in Supplementary Table 1. No informed consent and institutional ethical approval if the patients were required because all analyses were completed based on published data.

### Information Sources

We conducted a systematic literature search in PubMed, EMBASE, and CENTRAL from their inception until to May 17, 2020, and the latest search was updated on May 28, 2021. No restriction on language was imposed. The following terms were used to construct search strategy based on principle of combination of medical subject heading (MeSH) and text words: “ventilator weaning,” “spontaneous breathing trial,” “artificial respiration,” “random,” and various SBT techniques. Details of electronic search strategies and results identified are summarized in Supplementary Table 2. Any disagreements about study retrieval were solved based on consensus between two authors.

### Study Selection

All identified potentially eligible records were firstly imported into EndNote to develop a literature database, and then duplicate records were automatically eliminated by software. In the next step, two authors (LJY and XT) independently evaluated eligibility of unique records through screening titles and abstracts. Finally, they retrieved full-texts of all potentially relevant studies for further checking eligibility. To avoid literature omissions, clinical trial registry (such as www.clinicaltrials.gov) was also searched for unpublished and undergoing trials. Moreover, reference lists of included studies and relevant reviews were also manually screened to identify additional studies. Any controversies were solved based on consensus or adjudication with a third author (MC).

### Selection Criteria

For inclusion, a study should meet the following criteria: (a) enrolled adult patients suffering from respiratory failure who received invasive MV for at least 24 h regardless of gender; (b) compared at least two SBT techniques (T-piece, CPAP, ATC, or PSV); (c) reported at least one of the following outcomes including weaning success, reintubation, SBT success, duration of acute care, and ICU mortality; (d) used a RCT design with full-text. Moreover, abstract with sufficient information was also considered. A study was excluded if it covered at least one of the following criteria: (a) evaluated SBT methods in tracheostomized patients or in patients receiving noninvasive ventilation; (b) SBTs was only used as a part of the comprehensive weaning strategy; (c) with insufficient information and additional data cannot be added from authors; (d) used ineligible study design such as crossover design, quasi-randomized trials, observational studies, and commentary; and (e) duplicate study with poor methodology and insufficient data.

### Definition of Outcome

Our primary outcome was weaning success, which was defined as the absence of reintubation and/or resumption of ventilatory support for 48 h after extubation (45, 94). Secondary outcomes included reintubation rate (which was defined as the rate of reintubation within 48 h following extubation) (45, 94), successful SBT (if the patient showed no signs of intolerance when the SBT was performed, the SBT was considered successful) (45, 94), duration of ICU or long-term weaning unit (defined as the time from randomization to ICU or LWU) (46), and ICU mortality (defined as rate of the number of deaths during staying in ICU was divided by the number of all patients) (46).

### Data Extraction

Two authors independently extracted the following relevant information from eligible studies with a predesigned standard information extraction sheet:(a) details of the studies including the first author's name, publication year, country, publication type, study design, types of intervention and control;(b) population characteristics including ventilation time before SBT, age, and severity of the disease; (c) reported outcomes including primary and secondary outcomes. What's more, we also extracted the information about quality of included studies. Discrepancies were resolved through consulting a third author. Leading author was contacted *via* email if the information of interest is absent.

### Risk of Bias Assessment

Two independent authors assessed the methodological quality by using the Cochrane risk of bias assessment tool from the following seven items (47, 48): random sequence generation, allocation concealment, blinding of participants and personnel, blinding of outcome assessors, incomplete outcome data, selective reporting, and other bias. Each item was labeled as low, unclear, or high risk of bias according to the evaluation criteria (47). Among these target outcomes, all except for two (ICU mortality and ICU duration) depended on subjective judgement, which means the existence of different detection bias; therefore we performed risk of bias assessment respectively. We usually assume that blinding of outcome assessment was generally low risk of bias for objective outcomes.

### Geometry of the Network

Network plots were produced to visualize the body of available evidence. In network geometry, each node represents a treatment and each line between the nodes represents a direct comparison. The size of the nodes and the thickness of the lines are proportional to total sample size and precision, respectively.

### Statistical Analysis

All analyses were done using RevMan 5.3 (used for pairwise meta-analysis) and R version 3.6.1 (used for conducting NMA with gemtc package, assessing global heterogeneity, and calculating the surface under the cumulative ranking curve [SUCRA]) and STATA version 15.0 (used for estimation of inconsistency and local heterogeneity, funnel plot, and contribution plot).

#### Methods for Direct Treatment Comparisons

We conducted a pairwise meta-analysis for all comparisons by using the DerSimonian–Laird (DL) random-effects model. Odds ratio (OR) with 95% confidence interval (CI) was calculated for dichotomous outcome, whereas standardized mean difference with 95% CI was calculated for continuous outcome. We used Chi square and *I*^2^ statistic simultaneously to evaluate the heterogeneity across studies. *I*^2^ statistic measures the proportion of the overall variation that is attributable to between-study heterogeneity and *I*^2^ ≥ 50% was deemed as substantial heterogeneity (49, 50). For studies with multiple arms, outcome data were extracted from each group that meets the inclusion criteria, and then were created independent pairwise comparisons (43).

#### Methods for Indirect and Mixed Comparisons

For each endpoint, a Bayesian random-effects NMA (51, 52) was conducted to combine direct and indirect results. We calculated the relative ranking probabilities of being the best, second best for each weaning method, and so on. What's more, we also employed the SUCRA to estimate the ranking probabilities for available weaning methods on various outcomes (53). When one weaning technique is regarded as the best one without uncertainty, SUCRA value equals 1. If not, we draw an opposite conclusion (53, 54).

#### Assessment of Consistency and Heterogeneity

To explore the inconsistency of the entire network, the design-by treatment interaction model was used (55, 56). By using the “ifplot” command, inconsistency factor (IF) was calculated in each closed loop (a loop is made up of three technologies) to estimate the local inconsistencies, with values near 1 denoting statistical consistency (57, 58). Besides, a node-splitting method was undertaken to assess the potential inconsistency between the direct and indirect evidence for each comparison, which is a node in a direct acyclic graph (59). A *P* of more than 0.05 was deemed as consistent, which implied that the information from both sources of evidence contains enough similarities to be combined (60). A global heterogeneity was quantified using the *I*^2^-statistic. The prediction intervals for the pooled ORs provided a limited range in which the relative effect of a future similar study is expected to be involved (61, 62). The predictive interval plot, considering the extent of heterogeneity, was used to assess the magnitude of uncertainty in the estimated effect size for the NMA (63). If uncertainty is affected by heterogeneity, discordances exist between the confidence intervals of relative treatment effects and their predictive intervals.

### Contribution Plot and Publication Bias

A contribution plot revealed the influence of each direct comparison to the estimation of the network summary effects, which helped to make an objective appraisal of the overall quality of evidence from NMA (58, 64). A comparison-adjusted funnel plot was constructed to inspect the small-study effects when sufficient number of eligible studies were analyzed in a single pair of comparison (<10) (65).

### GRADE Evaluation on Quality of Evidence

We evaluated the certainty of evidence contributing to all network estimates of the primary outcomes by using the Grading of Recommendations Assessment, Development, and Evaluation (GRADE) framework (66). Disagreements„ if any, were resolved by consulting a third researcher.

## Results

### Study Selection and Characteristics

**Figure 1:**
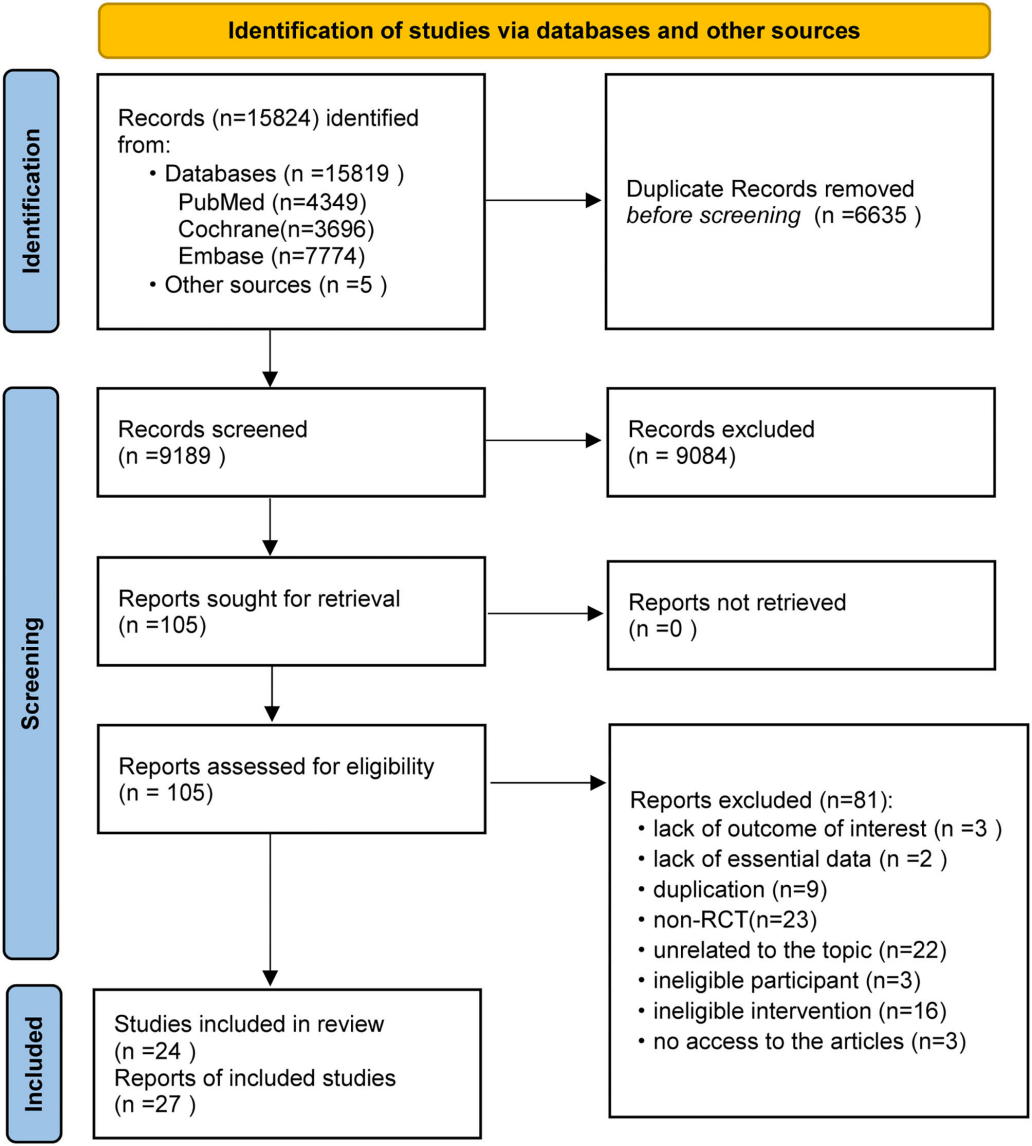
PRISMA flow diagram of retrieval and selection of studies.

After assessment of 105 full-text articles, 24 publications involving 4,241 subjects were included to investigate the efficacy of T-piece, PSV, CPAP, and ATC in critically ill patients weaning from MV (21, 22, 30–40, 45, 67–75, 94). We designed Figure 1 to outline the details of capturing and selecting studies.

**Figure 2:**
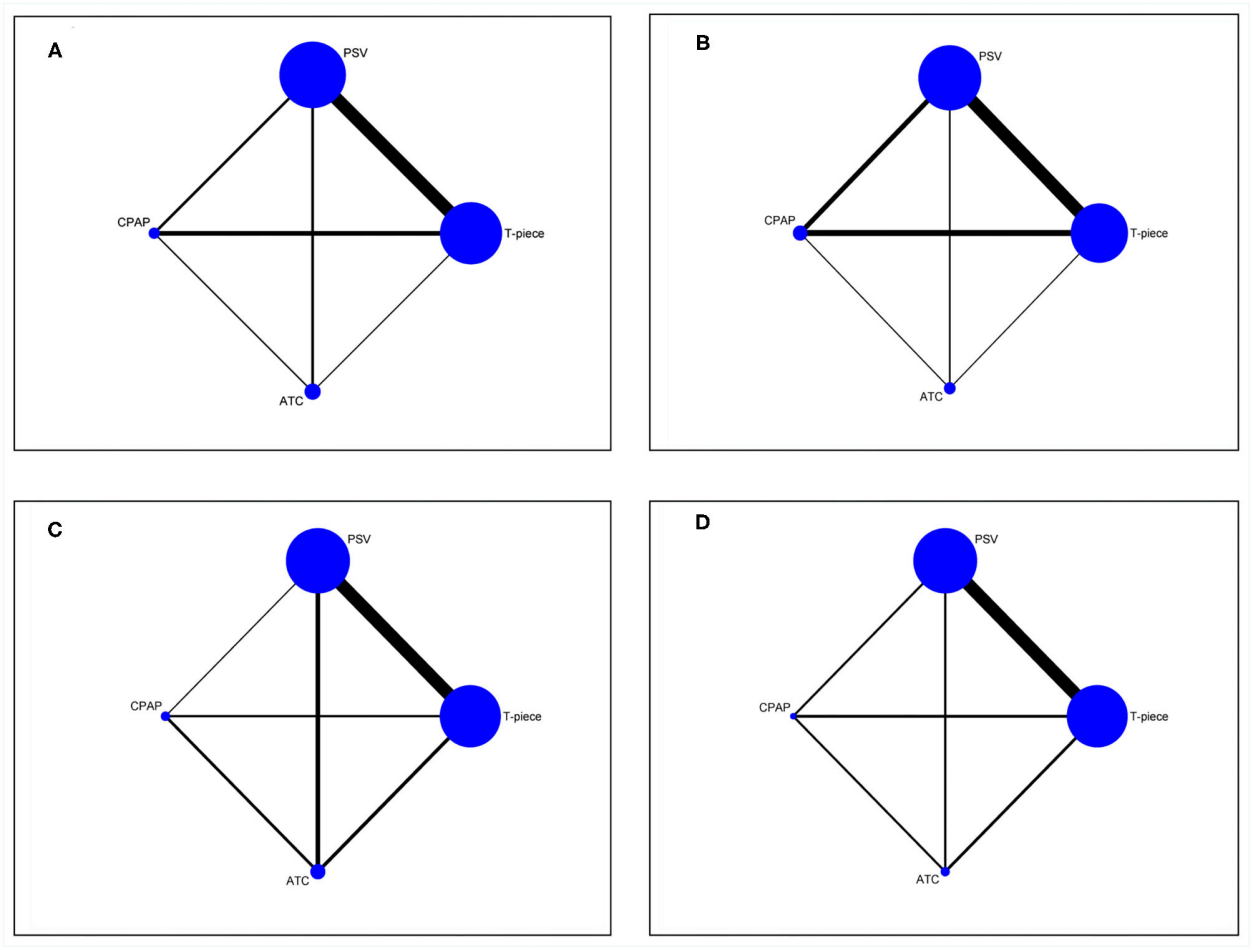
Evidence structure of eligible comparisons for network meta-analysis. **(A)** weaning success. **(B)**. reintubation. **(C)** SBT success. **(D)** ICU or LWU length of stay. All SBT techniques are represented as blue solid circles, and existing head-to-head (direct) comparisons are drawn as black solid lines. The size of every node is proportion to the number of randomly assigned participants (sample size) and the width of the lines is proportion to the number of RCTs for each pairwise comparison. PSV, pressure support ventilation; CPAP, continuous positive airway pressure; ATC, automatic tube compensation.

The baseline characteristics of included articles are summarized in Supplementary Table 3. The majority of the studies were designed double-arm trials (21/24, 87.5%) (21, 30, 31, 33–37, 39, 40, 45, 67–75, 94). Publication year was between 1991 and 2020, and the number of participants of individual study ranged from 14 to 578. To illustrate the head-to-head comparisons involved in the NMA, network plots for four outcomes were delineated in Figure 2. T-piece (20 studies) (21, 22, 31–33, 35, 37–40, 45, 67–73, 75, 94) and PSV (20 studies) (22, 31–33, 35–39, 45, 67, 69–75, 94) were the most frequently investigated SBT methods, whereas CPAP (six studies) (21, 22, 30, 32, 34, 38, 40, 68) and ATC(six studies) (32, 34, 36, 74) acquired fewer samples, thus suggesting a higher potential deviation in traditional meta-analysis.

### Methodological Quality of Studies

Out of 24 RCTs, seven (29.1%) (32, 33, 35, 36, 45, 74, 75) did not describe the method of generating random sequence. Eight RCTs (33.3%) (33, 35, 36, 38, 39, 71, 74, 75) did not report the details of allocation concealment, which could cause potential selection bias. Besides, one study (34) stated that personnel supervising of the SBTs failed to conceal allocation, and was therefore considered to present a high risk of bias. For subjective outcomes (weaning success, reintubation, and SBT success), eight studies (21, 22, 30, 31, 34, 38, 68, 94) provided details on blinding of outcome assessors, and three articles (37, 67, 71) did not evaluate outcomes in a blinded manner. Since all studies stated a clear patient flow or used intention-to-treat analysis, there was no hint of attrition bias. What's more, no study selectively reported results. Risk of bias summary was documented in Supplementary Table 4.

### Weaning Success

The effects of four extubation strategies on weaning success from pairwise metaanalyses can be found in Figure 2A. Among six direct comparisons in direct random-effects meta-analysis, ATC was associated with increased weaning success rate compared with T-piece (OR, 0.28; 95% CI, 0.13 to 0.64) and PSV (OR, 0.53; 95% CI, 0.32 to 0.88), respectively. Remaining comparisons were not statistically significant (see Supplementary Figure 1).

**Figure 3:**
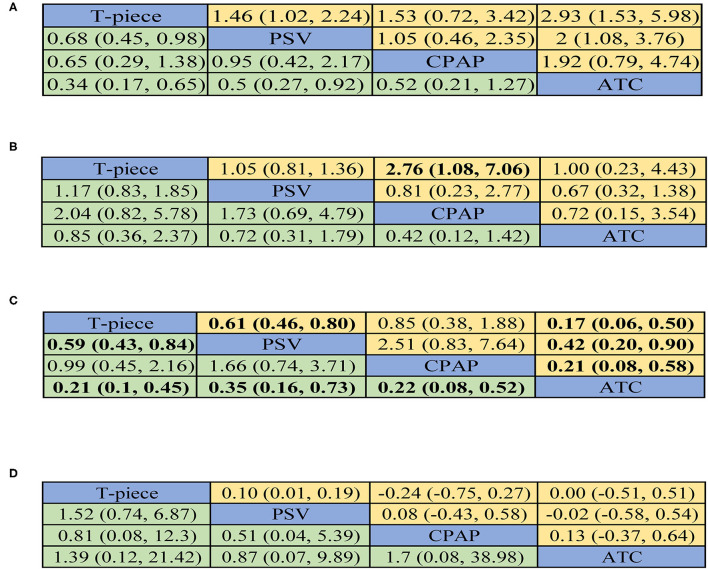
Summary for four outcomes of different SBT techniques. **(A)** weaning success. **(B)** reintubation. **(C)** SBT success. **(D)**. ICU or LWU length of stay. If available, the upper right half presented results from pairwise meta-analysis and the left lower half showed the results from network meta-analysis. For direct comparison, odds ratios (ORs) below 1 favor the row-defining treatment. For indirect comparison, ORs below 1 favor the column-defining treatment. For numerical data, the number in each cell represented the effect size of the treatment in upper left area minus the treatment in bottom right area. Significant results are in bold print. PSV: pressure support ventilation, CPAP: continuous positive airway pressure, ATC: automatic tube compensation.

In NMA, ATC was superior to the T-piece (OR, 0.34; 95% CI, 0.17 to 0.65) and PSV (OR, 0.5; 95% CI, 0.27 to 0.92) in terms of weaning success, respectively. Besides, an improvement effect of weaning success was detected for the comparison between PSV and T-piece (OR, 0.68; 95% CI, 0.45 to 0.98). Figure 3A reported all pooled results of the NMA.

### Reintubation

Of all 24 eligible RCTs, 17 (21, 22, 30–32, 37–40, 45, 67–69, 71–73, 94) reported the reintubation within 48 h following extubation, which included six direct comparisons (Figure 2B). CPAP could slightly decrease reintubation compared with T-piece (OR, 2.76; 95% CI, 1.08 to 7.06). All pooled results from traditional meta-analysis can be found in Supplementary Figure 2.

In NMA, all comparisons did not show significant effects on reintubation. All pooled results can be found in Figure 3B.

### SBT Success

Of all eligible RCTs, 13 (21, 22, 32, 34, 39, 45, 67, 68, 70–73, 94) reported SBT success, which included six direct comparisons (Figure 2C). In all direct comparisons, the comparative efficacy of T-piece vs. PSV (OR, 0.61; 95% CI, 0.46 to 0.80), T-piece vs. ATC (OR, 0.17; 95% CI, 0.06 to 0.50), PSV vs. ATC (OR, 0.42; 95% CI, 0.20 to 0.90), and CPAP vs. ATC (OR, 0.21; 95% CI, 0.08 to 0.58) reached statistical significance. All pooled results from direct comparisons can be obtained in Supplementary Figure 3.

The results of comparisons of SBT success in our NMA are presented in Figure 3C. ATC exerted a trend of high SBT success when compared with T-piece (OR, 0.21; 95% CI, 0.1–0.45), PSV (OR, 0.35; 95% CI, 0.16–0.73), and CPAP (OR, 0.22; 95% CI, 0.08–0.52), respectively. PSV had significant superiority over T-piece in SBT success (OR, 0.59; 95% CI, 0.43–0.84).

### ICU or LWU Length of Stay

Of all included RCTs, seven (31, 32, 36, 39, 69–71) reported ICU or LWU length of stay, which included six direct-comparisons (Figure 2D). In all six direct-comparisons, no major differences between the four extubation technologies were observed (Supplementary Figure 4). In NMA, no significant difference was observed in any comparisons (Figure 3D).

### ICU Mortality

Of all 24 eligible studies, 10 RCTs (31, 35, 39, 45, 67, 69–71, 73, 94) which focused exclusively on T-piece and PSV investigated the ICU mortality. Direct evidence supports that there was no significant difference in the effect of PSV and T-piece (OR, 1.19; 95% CI, 0.89 to 1.59) without heterogeneity (*I*^2^ = 0%) (Supplementary Figure 5).

### Assessment of Consistency and Heterogeneity

The test of global inconsistency detected no significant difference between the consistency and inconsistency models for four outcomes (*P* = 0.690 for weaning success, *P* = 0.523 for reintubation, *P* = 0.951 for STB success, and *P* = 0.308 for ICU or LWU length of stay, respectively). For four outcomes, test for local inconsistency showed that all loops were consistent (Supplementary Figure 6). Predictive interval plot indicated 33.3%, 0.00%, 33.3%, and 0.00% of the comparisons for weaning success, reintubation, SBT success, and ICU or LWU length of stay respectively, and therefore no outcomes was substantially affected by the estimated heterogeneity in the network (Supplementary Figure 7). The common heterogeneity through the Bayesian meta-analysis was 0.224 for weaning success, 0.020 for reintubation, 0.036 for SBT success, and 0.000 for ICU or LWU length of stay.

### SUCRA and Ranking of all Treatments

We showed the mean values of SUCRA for providing the hierarchy ranking of different weaning technologies on weaning success, reintubation, SBT success, and ICU or LWU length of stay. According to SUCRA, T-piece ranked fourth, second, third, and second on increase of weaning success, reintubation, SBT success and ICU or LWU length of stay, among all strategies, with a probability of 85.2%, 51.7%, 49.8%, and 44.3%, respectively. Whereas ATC had a probability of 91.7%, 62.1%, 99.7% and 39.9% to rank first, first, first, and fourth for each corresponding outcome above (Supplementary Table 5). However, considering that the sample sizes of different interventions varied greatly, the results might be highly biased and should be interpreted with caution. The ranking of all SBT technologies is depicted in Supplementary Figure 8.

### Contribution Plot and Publication Bias

According to the contribution plots of the network (see Supplementary Figure 9), the comparison of T-piece (mode A) vs. PSV (mode B) or PSV (mode B) vs. ATC (mode D) in the four entire networks showed 26.4% and 24.3% for weaning success, 32.7% and 23.9% for reintubation, 31.0% and 18.5% for SBT success, 29.5% and 19.2% for ICU or LWU length of stay, respectively.

We performed comparison-adjusted funnel-plot analysis for four outcomes (Supplementary Figure 10). The funnel plots were relatively asymmetric, highlighting that there is a significant risk of publication bias in our study.

### GRADE Evaluation on Quality of Evidence

According to GRADE, the quality of evidence ranged from very low to high, but was rated as low and as very low for most comparisons. In terms of T-piece vs. PSV, the quality was low for ICU or LWU length of stay and weaning success, and was very low for SBT success and reintubation, whereas moderate for ICU mortality. Quality of evidence was low for the overall ranking of treatment for weaning success, reintubation, ICU or LWU length of stay, and SBT success (Supplementary Table 6).

## Discussion

### Summary of Main Findings

This is the first NMA on this topic. After completing all analyses, we obtained several important findings: (a) Evidence from direct and NMA showed that ATC obtained superior weaning success compared to T-piece and PSV. Besides, the direct evidence demonstrated patients receiving PSV (vs. T-piece) appeared to be more likely to be extubated successfully; (b) Direct evidence suggested that T-piece had higher reintubation rate vs. CPAP, but these findings were not be supported by network evidence; (c) Direct evidence indicated that ATC was superior to others in SBT success, PSV was also better than T-piece in terms of this given outcome, and all statistically significant findings were detected in network meta-analyses; (d) In terms of prolonging ICU or LWU length of stay, no weaning technologies have been shown superior to another which were determined both directly and thorough NMA; (e) Compared with T-piece, PSV did not show different effects on the ICU mortality, whereas this conclusion was supported by direct evidence only; (f) The ranking of all weaning modes was ATC, CPAP, PSV, and T-piece in enhancing weaning success; (g) For increasing SBT success, the ranking of all weaning modes was ATC, PSV, T-piece, and CPAP; (h) The ranking of all weaning modes was ATC, T-piece, PSV, and CPAP in terms of reintubation rate; and (i) For prolonging ICU or LWU length of stay, the ranking of all weaning modes was CPAP, T-piece, PSV, and ATC.

Automatic tube compensation is a new mode of ventilatory assistance. It potentially simulates spontaneous breathing without the endotracheal tube, and so it has been called as “electronic extubation” (76, 77). There are several possible explanations for this clinical observation that ATC might be more efficacious than other investigated SBT techniques performed before extubation in critical patients. First and foremost, according to the actual flow that assists the spontaneously breathing intubated patient (78), ATC gives dynamic pressure support during the breathing cycle, which can automatically compensate for the non-linear resistance added by the artificial airway (21, 76, 79). This characteristic of ATC causes a reduction in the work of breathing (17, 80), and thus increases the probability of successful extubation (81). Secondly, ATC is able to maintain the natural and variable breathing pattern to the greatest extent (82, 83), which can more closely represent the postextubation scenario. This potential advantage of ATC can improve synchronization between patient and ventilator, and then promote respiratory comfort (82, 84, 85). Meanwhile, it can result in more significant predictive values for successful weaning and extubation (23). Last but not least, as a result of auto-positive end expiratory pressure (PEEP), ineffective ventilator-triggering is more likely to be less common with ATC than with PSV (77). Hence, ATC is ideally suitable for the weaning process (24).

Though direct evidence suggested that T-piece had higher reintubation rate when compared with CPAP, this finding was not supported by network evidence. Since network evidence combined the direct and indirect evidence in the same analytical model and more eligible RCTs were included, these results were more reliable and accurate.

Pressure support ventilation is widely used to overcome the additional work of breathing and pressure–time product exerted by the endotracheal tubes (18, 22, 86). Consequently, it can significantly decrease the endocrine stress response and relieve the clinical picture of intolerance (37, 38, 87). Furthermore, PSV allows patients to control the respiratory rate and the inspiratory flow during the spontaneous inspiration, thereby diminishing the oxygen consumption of respiratory muscles and preventing fatigue (88–90). These may be the primary reasons why PSV SBTs result in both higher SBT and extubation success rates compared with a T-piece SBT. This finding is broadly in line with previous work. A moderate-quality evidence (91) demonstrated that some intubated subjects who previously failed a weaning trial through the T-tube but continued a weaning trial with PSV were extubated successfully. A latest large-scale multicenter trial also compared PSV and T-piece ventilation in adults and noted that PSV SBT produced significantly higher rates of successful extubation, not adversely influencing reintubation rates (70).

### Agreements and Disagreements in the Current Literature

It was worth mentioning that several studies have exclusively investigated the efficacy and safety of at least two modalities of ventilator weaning, but primary studies comparing all the approaches have but one and cannot identify subtle clinical differences due to small sample size. To date, three traditional pairwise metaanalyses with full-text have been performed to evaluate the comparative efficacy of PSV vs. T-piece (46, 92)and PSV vs. other alternative SBT techniques (41) in patients ready to be liberated from MV. However, no head-to-head meta-analysis comparing all SBTs with each other has been reported. Consequently, that in which SBT technique is superior remains to be elucidated.

The results of Ladeira et al. (46) indicated an improvement in PSV group for successful SBTs among patients with simple weaning, but no difference between these two strategies for weaning success, ICU mortality, reintubation, ICU and LWU length of stay was found. Li et al. (92) found no difference between PSV SBT mode and T-piece SBT mode in all outcomes reported in the above-mentioned trial. Burns et al. (41)verified that extubation only tended to be more successful during PSV as compared with T-piece, but there was no difference between PSV vs. CPAP and PSV vs. ATC. After excluding an outlier trial, authors observed that patients undergoing PSV are more likely to pass an SBT. In contrast to previous metaanalyses, we comprehensively evaluated four common SBT technologies and obtained more informative findings. Firstly, we found that PSVs were associated with higher weaning success and SBT success, which is in agreement with previous results, but only these findings were confirmed by network metaanalyses. In addition, our analysis supported that ATC is an important weaning alternative for critically ill patients. Without increasing the reintubation rate and ICU or LWU length of stay, ATC provides clinical benefits in improving weaning success and SBT success. We also firstly make hierarchies of four different SBT technologies including T-piece, PSV, CPAP, and TAC, all of which were not reported in previous studies.

### Strengths and Limitations

Our NMA has certain important strengths including (a) We designed comprehensive search algorithms to obtain and identified eligible studies in critically ill patients, thereby minimizing information bias and enhancing generalizability; (b) NMA method allowed us to assess the results from both direct comparison and mixed-treatment comparisons, and thus optimally addressing the relative effectiveness of those SBT techniques; (c) We just included RCTs, which were the highest level of evidence; so we deemed that our pooled results can reflect closely the true effectiveness of the four most commonly performed SBT modes; and (d) We rated the certainty of evidence by the GRADE approach when explaining each unique comparison and across the network.

Nevertheless, some limitations in this study merited further discussion, including (a) Due to paucity of available data, we introduced criteria for pooling ventilation techniques. Many of the trials included varied in the level of pressure or did not specify whether PEEP was added; however, when implementing similar weaning strategies, we considered them to be in a clinically similar condition and combined them into a single group. This action may induce potential heterogeneity. (b) Since few publications existed, it is impossible to assess the impact of the mode of ventilation on other important indicators, such as hospital length of stay, hospital mortality, total duration of MV, and adverse events. Currently, most of the researchers monitored patients only during ICU stay, and very little data was available when they moved into the general ward. Further studies with a larger number of patients are warranted to consider these problems to gain full insight into the real effect of various extubation strategies. (c) No trials were designed to evaluate the impact of ATC and CPAP on ICU mortality in present. Also, we only captured 10 RCTs by directly comparing PSV and T-piece focusing on this parameter; thus larger studies with excellent designs are warranted to make up the gap. (d) It is important that neither patients nor personnel could be blinded after randomization as different SBT technologies had different requirements at the different preparation stages. We believe that this factor has potential influence on the results. However, the majority of weaning and extubation studies were not free from this limitation. (e) We did not specifically stratify all interventions in the current study, which may introduce a potential bias. However, the major aim of this NMA is to generally determine the comparative efficacy and safety of available macroscopic SBT techniques. Certainly, we suggest conducting future studies to further specifically differentiate the efficacy and safety of different regimes (e.g., low, middle, or high PSV) of each SBT technique.

### Implications for Further Research

Spontaneous breathing trials are an integrated component of the weaning assessment, so the “weaning condition” of a patient entering to SBT will influence the accuracy of different SBT methods. On the basis of the difficulty and duration of the weaning process, patients are divided into three categories: simple weaning, difficult weaning, and prolonged weaning (12, 93). In this review, the target patients in most of the studies included belonged to simple weaning, and our analysis supported the selection of ATC as an important alternative for this group. Hence, if one method to perform SBT has any superiority over the other, improvement in weaning outcome is more likely to be expected in selected populations at higher risk for prolonged weaning and difficult weaning. Further studies should be conducted to establish this classification and to confirm how related clinical outcomes are affected in each category of weaning, finalizing the optimal weaning strategy in specific weaning situations. Meanwhile, researchers should pay more attention to ATC weaning mode to clarify its role in weaning patients off mechanical ventilation.

It must be noted that, as an objective marker of identifying the severity, the MV duration before conducting SBT can reflect the demands for ventilation, the risk of suffering from infection, and refractory bronchospasm, all of which were positively associated with worse prognosis. A previous study (69) has revealed that the MV duration before conducting SBT may greatly increase the risk of weaning failure within 48 h. However, the role of this factor under the different SBT modes (PSV, T-piece, CPAP, and ATC) and among specific populations is unclear, which should be further clarified in future studies.

## Conclusions

In summary, the present NMA demonstrated that ATC is an alternative mode of ventilation for critically ill patients. Our finding should be interpreted with caution as it generates from RCT with small sample sizes. Further large scale and well-designed studies are needed to confirm this point.

## Data Availability Statement

The original contributions presented in the study are included in the article/Supplementary Material, further inquiries can be directed to the corresponding author.

## Author Contributions

L-JY, XT, MC, and MJ-H: conception and design. XT and MJ-H: administrative support. L-JY, XT, and MC: provision of study materials or patients. L-JY, XT: collection and assembly of data. L-JY and XT: data analysis and interpretation. L-JY, XT, MC, J-ML, NX, and MJ-H: manuscript writing and final approval of manuscript. All authors contributed to the article and approved the submitted version.

## Conflict of Interest

The authors declare that the research was conducted in the absence of any commercial or financial relationships that could be construed as a potential conflict of interest.

## Publisher's Note

All claims expressed in this article are solely those of the authors and do not necessarily represent those of their affiliated organizations, or those of the publisher, the editors and the reviewers. Any product that may be evaluated in this article, or claim that may be made by its manufacturer, is not guaranteed or endorsed by the publisher.
