# Supplementary material for: Comparative Efficacy and Safety of Four Different Spontaneous Breathing Trials for Weaning From Mechanical Ventilation: A Systematic Review and Network Meta-Analysis
Source: Front Med (Lausanne). 2021 Nov 22;8:731196. doi: 10.3389/fmed.2021.731196 (PMC8647911; doi:10.3389/fmed.2021.731196)
Supplement: Supplementary file 1 [file Data_Sheet_1.PDF]

Supplementary Materials

Contents of supplementary materials

Table S1. PRISMA-NMA checklist 3
Table S2. Search strategies and results of databases 9
Table S3. Characteristics of the 24 studies included in the network Meta-analysis. 13
Table S4. Risk of bias summary of included studies 19
Figure S1. Results of direct pairwise meta-analysis for weaning success错误!未定义书签。1
Figure S2. Results of direct pairwise meta-analysis for reintubation.........错误!未定义书签。
Figure S3. Results of direct pairwise meta-analysis for SBT success........错误!未定义书签。
Figure S4. Results of direct pairwise meta-analysis for ICU or LWU length of stay错误!未定义书签。
Figure S5. Results of direct pairwise meta-analysis for ICU mortality .....错误!未定义书签。
Figure S6. Evaluation of inconsistency for four outcomes 26
Figure S7. Predictive intervals plot for four outcomes 2727
Table S5. Ranking probabilities of different SBT technologies on four outcomes 28
Figure S8. Ranking of all SBT technologies 29
Figure S9. Contribution plot for four outcomes 3030
Figure S10. Comparison-adjusted funnel plot for four outcomes 311
Table S6. Evaluation of the quality of evidence 3232

Table S1. PRISMA-NMA Checklist

Section/Topic	Item #	Checklist Item	Reported on Page #
**TITLE**			
Title	1	Identify the report as a systematic review *incorporating a network meta-analysis (or related form of meta-analysis).*	1
**ABSTRACT**			
Structured summary	2	Provide a structured summary including, as applicable:<br>**Background:** main objectives<br>**Methods:** data sources; study eligibility criteria, participants, and interventions; study appraisal; and *synthesis methods, such as network meta-analysis.*<br>**Results:** number of studies and participants identified; summary estimates with corresponding confidence/credible intervals; *treatment rankings may also be discussed. Authors may choose to summarize pairwise comparisons against a chosen treatment included in their analyses for brevity.*<br>**Discussion/Conclusions:** limitations; conclusions and implications of findings.<br>**Other:** primary source of funding; systematic review registration number with registry name.	2
**INTRODUCTION**			
Rationale	3	Describe the rationale for the review in the context of what is already known*, including mention of why a network meta-analysis has been conducted.*_	3
Objectives	4	Provide an explicit statement of questions being addressed, with reference to participants, interventions, comparisons, outcomes, and study design (PICOS).	3
**METHODS**			
Protocol and registration	5	Indicate whether a review protocol exists and if and where it can be accessed (e.g., Web address); and, if available, provide registration information, including registration number.	NA

Eligibility criteria	6	Specify study characteristics (e.g., PICOS, length of follow-up) and report characteristics (e.g., years considered, language, publication status) used as criteria for eligibility, giving rationale. *Clearly describe eligible treatments included in the treatment network, and note whether any have been clustered or merged into the same node (with justification).*_	5, Appendix 3
Information sources	7	Describe all information sources (e.g., databases with dates of coverage, contact with study authors to identify additional studies) in the search and date last searched.	4, Appendix 2
Search	8	Present full electronic search strategy for at least one database, including any limits used, such that it could be repeated.	4, Appendix 2
Study selection	9	State the process for selecting studies (i.e., screening, eligibility, included in systematic review, and, if applicable, included in the meta-analysis).	4-5
Data collection process	10	Describe method of data extraction from reports (e.g., piloted forms, independently, in duplicate) and any processes for obtaining and confirming data from investigators.	6
Data items	11	List and define all variables for which data were sought (e.g., PICOS, funding sources) and any assumptions and simplifications made.	6
**Geometry of the network**	**S1**	Describe methods used to explore the geometry of the treatment network under study and potential biases related to it. This should include how the evidence base has been graphically summarized for presentation, and what characteristics were compiled and used to describe the evidence base to readers.	6
Risk of bias within individual studies	12	Describe methods used for assessing risk of bias of individual studies (including specification of whether this was done at the study or outcome level), and how this information is to be used in any data synthesis.	6
Summary measures	13	State the principal summary measures (e.g., risk ratio, difference in means). *Also describe the use of additional summary measures assessed, such as treatment rankings and surface under the cumulative ranking curve (SUCRA) values, as well as modified approaches used to present summary*	7

		findings from meta-analyses.	
Planned methods of analysis	14	Describe the methods of handling data and combining results of studies for each network meta-analysis. This should include, but not be limited to:<br>• *Handling of multi-arm trials;*<br>• *Selection of variance structure;*<br>• *Selection of prior distributions in Bayesian analyses; and*<br>• *Assessment of model fit.*	7
**Assessment of Inconsistency**	**S2**	Describe the statistical methods used to evaluate the agreement of direct and indirect evidence in the treatment network(s) studied. Describe efforts taken to address its presence when found.	7-8
Risk of bias across studies	15	Specify any assessment of risk of bias that may affect the cumulative evidence (e.g., publication bias, selective reporting within studies).	8
Additional analyses	16	Describe methods of additional analyses if done, indicating which were pre-specified. This may include, but not be limited to, the following:<br>• Sensitivity or subgroup analyses;<br>• Meta-regression analyses;<br>• *Alternative formulations of the treatment network; and*<br>• *Use of alternative prior distributions for Bayesian analyses (if applicable).*_	NA
**RESULTS†**			
Study selection	17	Give numbers of studies screened, assessed for eligibility, and included in the review, with reasons for exclusions at each stage, ideally with a flow diagram.	8, Figure 1
**Presentation of network structure**	**S3**	Provide a network graph of the included studies to enable visualization of the geometry of the treatment network.	8, Figure 2
**Summary of network geometry**	**S4**	Provide a brief overview of characteristics of the treatment network. This may include commentary on the abundance of trials and randomized patients for the different interventions and pairwise comparisons in the network, gaps of evidence in the treatment network, and potential biases reflected by the network structure.	8-9, Figure 2, Appendix 3, Appendix 6

Study characteristics	18	For each study, present characteristics for which data were extracted (e.g., study size, PICOS, follow-up period) and provide the citations.	8-9, Appendix 3
Risk of bias within studies	19	Present data on risk of bias of each study and, if available, any outcome level assessment.	9, Appendix 4
Results of individual studies	20	For all outcomes considered (benefits or harms), present, for each study: 1) simple summary data for each intervention group, and 2) effect estimates and confidence intervals. *Modified approaches may be needed to deal with information from larger networks.*	9-11, Figure 3, Appendix 5
Synthesis of results	21	Present results of each meta-analysis done, including confidence/credible intervals. *In larger networks, authors may focus on comparisons versus a particular comparator (e.g. placebo or standard care), with full findings presented in an appendix. League tables and forest plots may be considered to summarize pairwise comparisons.* If additional summary measures were explored (such as treatment rankings), these should also be presented.	9-11, Figure 3, Appendix 7-8
**Exploration for inconsistency**	**S5**	Describe results from investigations of inconsistency. This may include such information as measures of model fit to compare consistency and inconsistency models, *P* values from statistical tests, or summary of inconsistency estimates from different parts of the treatment network.	11, Appendix 6
Risk of bias across studies	22	Present results of any assessment of risk of bias across studies for the evidence base being studied.	12, Appendix 11
Results of additional analyses	23	Give results of additional analyses, if done (e.g., sensitivity or subgroup analyses, meta-regression analyses*, alternative network geometries studied, alternative choice of prior distributions for Bayesian analyses,* and so forth).	NA
**DISCUSSION**			
Summary of evidence	24	Summarize the main findings, including the strength of evidence for each main outcome; consider their relevance to key groups (e.g., healthcare providers, users, and policy-makers).	12-14
Limitations	25	Discuss limitations at study and outcome level (e.g., risk of bias), and at review level (e.g., incomplete retrieval of identified research, reporting bias). *Comment on the validity of the*	14-15

		assumptions, such as transitivity and consistency. Comment on any concerns regarding network geometry (e.g., avoidance of certain comparisons).	
Conclusions	26	Provide a general interpretation of the results in the context of other evidence, and implications for future research.	16
**FUNDING**			
Funding	27	Describe sources of funding for the systematic review and other support (e.g., supply of data); role of funders for the systematic review. This should also include information regarding whether funding has been received from manufacturers of treatments in the network and/or whether some of the authors are content experts with professional conflicts of interest that could affect use of treatments in the network.	17

PICOS = population, intervention, comparators, outcomes, study design.

* Text in italics indicateS wording specific to reporting of network meta-analyses that has been added to guidance from the PRISMA statement.

† Authors may wish to plan for use of appendices to present all relevant information in full detail for items in this section.

Table S2. Search strategies and results of databases

Recent queries in pubmed	
Search	Query
#15	#6 AND #13 AND #14
#14	Search random* Sort by: PublicationDate
#13	#7 AND #8 AND #9 AND #10 AND #11 AND #12
#12	Search(automatic tube compensation*) OR Automatic endotracheal tube compensation Sort by: PublicationDate
#11	Search ((((CPAP Ventilation[Title/Abstract]) OR Continuous Positive Airway Pressure[Title/Abstract]) OR nCPAP Ventilation[Title/Abstract]) OR Airway Pressure Release Ventilation[Title/Abstract]) OR APRV Ventilation Mode*[Title/Abstract] Sort by: PublicationDate
#10	Search "Continuous Positive Airway Pressure"[Mesh] Sort by: PublicationDate
#9	Search(T-piece) OR T-Tube Sort by: PublicationDate
#8	Search (((((Positive Pressure Respiration[Title/Abstract]) OR Positive-Pressure Respirations[Title/Abstract]) OR Positive-Pressure Ventilation*[Title/Abstract]) OR Positive Pressure Ventilation[Title/Abstract]) OR Positive End-Expiratory Pressure*[Title/Abstract]) OR Positive End Expiratory Pressure[Title/Abstract] Sort by: PublicationDate
#7	Search "Positive-Pressure Respiration"[Mesh] Sort by: PublicationDate
#6	#1 OR #2 #3 OR #4 AND #5
#5	Search((Artificial Respiration*[Title/Abstract]) OR Mechanical Ventilation*[Title/Abstract]) OR pressure support[Title/Abstract] Sort by: PublicationDate
#4	Search "Respiration, Artificial"[Mesh] Sort by: PublicationDate
#3	Search spontaneous breathing trial* Sort by: PublicationDate
#2	Search(Respirator Weaning[Title/Abstract]) OR Mechanical Ventilator Weaning[Title/Abstract] Sort by: PublicationDate
#1	Search "Ventilator weaning"[Mesh] Sort by: PublicationDate

Embase search strategy

....

No. Query Results

#15. #7 AND #13 AND #14

#14. 'random*':ti,ab,kw

#13. #8 OR #9 OR #10 OR #11 OR #12

#12. 'automatic tube compensation':ti,ab,kw

#11. 'automatic tube compensation'/exp

#10. 'T-piece':ti,ab,kw OR 'T-tube':ti,ab,kw

#9. 'constant positive pressure breathing':ti,ab,kw OR 'positive-pressure respiration':ti,ab,kw OR 'continuous positive airway pressure':ti,ab,kw OR 'continuous positive pressure breathing':ti,ab,kw OR cpap:ti,ab,kw OR cppb:ti,ab,kw OR cppv:ti,ab,kw OR 'hyperbaric respiration':ti,ab,kw OR 'hyperbaric ventilation':ti,ab,kw OR 'positive pressure breathing':ti,ab,kw OR 'positive pressure ventilation':ti,ab,kw OR ' positive pressure ventilation mode':ti,ab,kw OR 'pressure respiration':ti,ab,kw OR 'pressure breathing':ti,ab,kw OR peep:ti,ab,kw OR 'positive endexpiratory pressure breathing':ti,ab,kw OR 'positive pressure ventilation':ti,ab,kw OR 'positive pressure ventilation model':ti,ab,kw OR 'pressure respiration':ti,ab,kw

#8. 'positive end expiratory pressure'/exp

#7. #1 OR #2 OR #3 OR #4 OR #5 OR #6

#6. 'respirator weaning':ti,ab,kw OR 'weaning of ventilation':ti,ab,kw OR 'ventilation weaning':ti,ab,kw OR 'wean off mechanical respiration':ti,ab,kw OR 'wean off respiratory support':ti,ab,kw OR 'wean off vent support':ti,ab,kw OR 'weaning from artificial respiration':ti,ab,kw OR 'weaning from mechanical ventilation':ti,ab,kw OR 'weaning from respirator':ti,ab,kw OR 'weaning of artificial respiration':ti,ab,kw OR 'weaning off mechanical ventilation':ti,ab,kw OR 'weaning off the respirator':ti,ab,kw OR 'weaning off the ventilator':ti,ab,kw

#5. 'ventilator weaning'/exp

#4. 'artificial respiration':ti,ab,kw OR 'controlled respiration':ti,ab,kw OR 'controlled ventilation':ti,ab,kw OR 'mechanical respiration':ti,ab,kw OR 'mechanical ventilation':ti,ab,kw

#3. 'artificial ventilation'/exp

#2. 'spontaneous breathing trial*':ti,ab,kw

#1. 'spontaneous breathing trial'/exp

....

Cochrane library search strategy

ID Search

#1 (spontaneous breathing trial*):ti,ab,kw

#2 MeSH descriptor: [Respiration, Artificial] explode all trees

#3 (Artificial Respiration*):ti,ab,kw OR (Mechanical Ventilation*):ti,ab,kw (Word variations have been searched)

#4 MeSH descriptor: [Ventilator Weaning] explode all trees

#5 (Respirator Weaning):ti,ab,kw OR (Mechanical Ventilator Weaning):ti,ab,kw (Word variations have been searched)

#6 #1 OR #2 OR #3 OR #4 OR #5

#7 MeSH descriptor: [Positive-Pressure Respiration] explode all trees

#8 (Positive Pressure Respiration):ti,ab,kw OR (Positive-Pressure Respirations):ti,ab,kw OR (Positive-Pressure Ventilation*):ti,ab,kw OR (Positive Pressure Ventilation):ti,ab,kw OR (Positive End-Expiratory Pressure*):ti,ab,kw OR (Positive End Expiratory Pressure):ti,ab,kw (Word variations have been searched)

#9 (T-piece):ti,ab,kw OR (T-tube):ti,ab,kw (Word variations have been searched)

#10 (Continuous Positive Airway Pressure):ti,ab,kw (Word variations have been searched)

#11 (Automatic tube compensation*):ti,ab,kw OR (Automatic endotracheal tube compensation):ti,ab,kw (Word variations have been searched)

#12 #7 OR #8 OR #9 OR #10 OR #11

#13 (random):ti,ab,kw (Word variations have been searched)

#14 #6 AND #12 AND #13

Table S3. Characteristics of the 24 studies included in the network Meta-analysis.

Author/year/ country	Publication type	Study design	Ventilation time before SBT	Reason for mechanical ventilation	Age (year)	Severity of disease	Interventions	Outcomes
Jones et al, 1991, USA	Full	Single center	NR	post-operation; chf/pulmonary; edema; sepsis; COPD/asthma; neurologic/sedation; airway protection; pneumonia; miscellaneous	T-piece: 64.60 ± 13.45<br>CPAP: 65.58 ± 16.71	NR	1.T-piece, ZEEP, 1 hour (n=52)<br>2.CPAP 5 cm H_2O, 1 hour (n=54)	①③
Esteban et al, 1997, Spain	Full	Multicenter	T-piece: 6 (4 – 9) days<br>PSV: 6 (4 - 12) days	neuromuscular disease, coma; COPD; acute lung injury	T-piece: 64 (53 – 71)<br>PSV: 64 (50 - 72)	SAPS II<br>T-piece: 36 (29 – 47)<br>PSV:36 (26 – 46)	1.T-piece, 2 hours (n=246)<br>2.PSV 7 cm H_2O, PEEP ≤ 5 cm H_2O, 2 hours (n=238)	①②③⑤
Koh et al, 2000, Korea	Full	Single center	T-piece: 314 ± 36 hours<br>PSV: 263 ± 57 hours	NA	T-piece: 65 ± 2<br>PSV: 55 ± 3	APACHE II<br>T-piece: 67 ± 5<br>PSV: 61 ± 3	1.T-piece,1 hour (n=22)<br>2.PSV 15 cm H_2O, decrease 5 cm H_2O/h, 30 minutes (n=20)	①③
Haberthur et al, 2002, Switzerland	Full	Single center	ATC: 142 ± 127 hours<br>PSV: 152 ± 142 hours<br>T-piece: 138 ± 0.95 hours	acute lung injury, miscellaneous, neurologic disorder, COPD	ATC: 59.4 ± 13.5<br>PSV: 54.5 ± 15.2<br>T-piece: 56.1 ± 14.0	APACHE II<br>ATC: 20.8 ± 6.4<br>PSV: 20.3 ± 7.3<br>T-piece: 19.6 ± 6.6	1.T-piece, 2 hours (n=30)<br>2.PSV 5 cm H_2O, PEEP 5 cm H_2O, 2 hours (n=30)<br>3.ATC, PEEP 5 cm H_2O, 2 hours (n= 30)	①⑤
Koksal et al, 2004, Turkey	Full	Single center	>48 hours	NA	T-piece: 75 ± 12<br>PSV: 73 ± 13	APACHE II<br>PSV: 18 ± 8	1.PSV ≤ 10 cm H_2O, FiO2 ≤ 0.4, PEEP ≤ 5 cm H_2O, 2 hours (n=20)	①③

					CPAP: 71 ± 16	CPAP: 17 ± 6<br>T-piece: 19 ± 6	2.CPAP ≤ 5 cm H_2O, FiO2 ≤ 0.4, 2 hours (n=20)<br>3.T-piece, 4 l/min, 2 hours (n=20)	
Matic et al, 2004, Croatia	Full	Single center	>48 hours	COPD; COMA; neuromuscular disease; acute pulmonary lesion;	T-piece: 49.5 (31.0 - 64.2)<br>PSV: 52.5 (26.1 - 61.4)	APACHE II<br>PSV: 26.0 (16.4 - 29.25)<br>T-piece: 24.3 (18 - 29)	1.T-piece, 2 hours (n=110)<br>2.PSV 8 cm H_2O, PEEP ≤ 5 cm H_2O, 2 hours (n=150)	①②③④⑤
Matic et al, 2007, Croatia	Full	Single center	T-piece: 124 (94 - 151) hours<br>PSV: 120 (88 - 139) hours	COPD	T-piece: 59 (41 - 71)<br>PSV: 57 (32 - 68)	APACHE II<br>T-piece: 29 (23 - 31)<br>PSV: 31 (23 - 34)	1.T-piece, 2 hours (n=31)<br>2.PSV 5 cm H_2O, 2 hours (n=32)	①②③④⑤
Cohen et al, 2009, Israel	Full	Single center	PSV: 6.3 ± 4.7 days<br>ATC: 5.9 ± 3.5 days	NA	PSV: 66.1 ± 18.1<br>ATC: 62.1 ± 16.6	APACHE II<br>PSV: 19.1 ± 7.0<br>ATC: 22.1 ± 7.9	1.PSV 7 cm H_2O, CPAP 5 cm H_2O (n=93)<br>2.ATC 100%, CPAP 5 cm H2O (n=87)	①③⑤
Figueroa-Casas et al, 2010, USA	Full	Single center	CPAP: 4.7 ± 3.2 days<br>ATC:5.1 ± 4.2 days	NA	CPAP: 50.8 ± 18.6<br>ATC: 51.7 ± 20.2	APACHE II<br>CPAP: 13.6 ± 5.7<br>ATC:13.9 ± 4.5	1.CPAP 5 cm H_2O 30min (n=62)<br>2.ATC 100%, PEEP 5 cm H_2O 30min (n=60)	①⑤
Molina-Saldarriaga et al, 2010, Colombia	Full	Multicenter	6 ± 3.34 days	COPD	T-piece: 63 ± 11<br>CPAP: 64.4 ± 13	APACHE II<br>T-piece: 14 (11 – 17,5)<br>CPAP: 15.5 (11 – 21)	1.T-piece,30 min (n=25)<br>2.CPAP 85% of intrinsic PEEP, 30 min (n=25)	①③⑤

Cekman et al, 2011, Turkey	Full	Single center	T-piece: 6.2 ± 5.0 days<br>CPAP: 7.2 ± 5.4 days	multitrauma; pneumonia; heart failure; sepsis with acute lung injury; neurological; after emergency surgery	T-piece: 67 ± 17<br>CPAP: 71 ± 15	APACHE II<br>T-piece: 29 ± 6<br>CPAP: 28 ± 7	1.T-piece, 4 L/min (n=20)<br>2.CPAP, PEEP ≤ 5 cm H_2O, FiO2 ≤ 0.4 (n=20)	①③
Vats et al, 2012, India	Full	Single center	>48 hours	COPD; acute respiratory failure	18-70	NR	1.T-piece, 2 hours (n=20)<br>2.PSV 7 cm H_2O, 2 hours (n=20)	①②③⑤
Lourenco et al, 2013, Brazil	Full	Single center	NR	NA	NR	NR	1.T-piece, 30 min (n=14)<br>2.PSV 10 cm H_2O, 30 min (n=14)	①③④⑤
Zhang et al, 2014, China	Full	Single center	T-piece: 4.46 ± 3.18 days<br>PSV: 4.84 ± 4.11 days	NA	T-piece: 71.71 ± 9.9<br>PSV: 70.95 ± 12.61	NR	1.T-piece 4L/min, 30min (n=115)<br>2.PSV 5 cm H_2O, PEEP 5 cm H_2O, FiO_2 30%, 30 min(n=93)	①
Zanfaly et al, 2014, Egypt	Full	Single center	>48 hours	postoperative; olytrauma; COPD; sepsis; miscillaneous	ATC: 46.2 ± 10.7<br>PSV: 47.1 ± 11.5<br>CPAP: 46.7 ± 12.0<br>T-piece:45.7 ± 12.7	APACHE II<br>ATC: 21.8 ± 5<br>PSV: 20.3 ± 7.3<br>CPAP: 24.4 ± 4.5<br>T-piece: 21.6 ± 6.6	1.ATC 100%, CPAP 5 cm H_2O, 2 hours (n=30)<br>2.PSV 7 cm H_2O, 2 hours (n=30)<br>3.CPAP 5 cm H_2O, 2 hours (n=30)<br>4.T-piece, 2 hours (n=30)	①③④⑤
Teixeira et al, 2015, Brazil	Full	Single center	T-piece: 7.1 ± 4.1 days<br>PSV: 6.6 ± 4.4 days	NA	T-piece:46.8 ± 20.8<br>PSV:44.3 ± 19.8	APACHE II<br>T-piece: 22.7 ± 4.2<br>PSV: 21.9 ± 5.4	1.T-piece, 30-90 min (n=66)<br>2.PSV 7 cm H_2O, PEEP 5-8 cm H_2O, FiO2 ≤ 45%, 30-90min (n=46)	①②③④⑤

Wafy et al, 2015, Egypt	Abstract	Single center	NR	NA	58.6 ± 12.3	NR	1.PSV (not specified) (n=88)<br>2.ATC (not specified) (n=78)	①
Vats et al, 2016, India	Full	Single center	>48 hours	NA	18-70	NR	1.T-piece, 2 hours (n=20)<br>2.PSV 7 cm H_2O, 2 hours (n=20)	①③⑤
Kashefi et al, 2017, Iran	Full	Single center	NR	NA	PSVa:57.6 ± 19.1<br>PSVb:52.6 ± 14.5<br>ATC:55.3 ± 18.9	NR	1.PSV 5 cm H_2O (n=35)<br>2.PSV 8 cm H_2O (n=35)<br>3.ATC(not specified) (n=35)	①
Chittawatanarat et al, 2018, Thailand	Full	Single center	T-piece: 26 (15.5 - 46) hours<br>PSV: 31 (18 - 60.5) hours	the hemodynamic, respiratory, and metabolic causes	T-piece: 56 (46 – 65) PSV: 56 (45 – 67)	APACHE II<br>T-piece: 11 (9 – 13)<br>PSV: 11 (9 – 13)	1.T-piece 10-15 L/min, 2 hours (n=260)<br>2.PSV 5-7 cm H_2O, PEEP 5 cm H_2O, 2 hours(n=260)	①②③④
Pellegrini et al, 2018, Brazil	Full	Multicenter	T-piece: 10.82 ± 9.1 days<br>PSV: 7.31 ± 4.9 days	exacerbation of COPD; pneumonia; central nervous system depression; post-operative acute decompensated heart failure; pulmonary embolism	U-piece: 67.99 ± 11.37<br>PSV: 67.30 ± 9.83	SAPS II<br>T-piece: 68.04 ± 12.36<br>PSV: 67 ± 14.28	1.T-piece, 30 min(n=99)<br>2.PSV 10cm H_2O, 30min(n=91)	①②③④
Subira et al, 2019, Spain	Full	Multicenter	T-piece: 4 (2-8) days<br>PSV:4 (2-8) days	NA	T-piece: 63 (53-73)<br>PSV: 65 (52-75)	APACHE II<br>T-piece: 16 (11 - 22)<br>PSV: 16 (11 - 22)	1.T-piece, 2 hours (n=578)<br>2.PSV 8 cmH_2O, 30 min (n=575)	②④⑤

Faleh et al, 2020, Africa	Abstract	Single center	NR	mainly a face neck burned	29±11	NR	1.T-piece, 30-120 min (n=15)<br>2.PSV 8 cm H_2O, PEEP 0 cm H_2O, 30-120 min (n=15)	①
Jamoussi et al, 2020, Africa	Abstract	Single center	T-piece: 6.87 ± 4.3 days<br>PSV: 6.06 ± 4.8 days	COPD	66 ± 10	NR	1.T-piece (n=15)<br>2.PSV (not specified) (n=17)	①

NR, not reported; COPD, chronic obstructive pulmonary disease; APACHE II, Acute Physiology and Chronic Health Evaluation II; SAPS II, simplified acute physiology score II; PEEP, positive end-expiratory pressure, ZEEP, zero end-expiratory pressure, PSV, pressure support; CPAP , continuous positive airway pressure; ATC, automatic tube compensation; ① to ⑤ represents weaning success, ICU mortality, reintubation, ICU or LWU length of stay, and successful spontaneous breathing trial respectively.

Figure S4. Risk of bias summary of included studies

Study ID	Random Sequence generation	Allocation concealment	Blinding of subjective outcome assessment	Blinding of objective outcome assessment	Incomplete outcome data	Selective reporting	Other bias
Cekman 2011	L	L	L	L	L	L	L
Chittawatanarat 2018	L	L	L	L	L	L	U
Cohen 2009	L	L	L	L	L	L	L
Esteban 1997	L	L	L	L	L	L	L
Faleh 2020	U	U	U	L	L	L	L
Figueroa-Casas 2010	L	H	L	L	L	L	L
Haberthur 2002	L	L	L	L	L	L	U
Jamoussi 2020	U	U	U	L	L	L	L
Jones 1991	L	L	U	L	L	L	L
Kashefi 2017	U	U	U	L	L	L	L
Koh 2000	L	L	H	L	L	L	U
Koksal 2004	L	U	L	L	L	L	L
Lourenco 2013	L	U	U	L	L	L	L
Matic 2004	U	L	U	L	L	L	L
Matic 2007	L	L	H	L	L	L	L
Molina-Saldarriaga 2010	L	L	L	L	L	L	L
Pellegrini 2018	L	L	U	L	L	L	L
Subira 2019	L	L	U	L	L	L	L
Teixeira 2015	L	U	H	L	L	L	L
Vats 2012	L	L	U	L	L	L	L
Vats 2016	L	L	U	L	L	L	L
Wafy 2015	U	U	U	L	L	L	L
Zanfaly 2014	U	L	U	L	L	L	U
Zhang 2014	U	U	U	L	L	L	L

L, low risk of bias; U, unclear risk of bias; H, high risk of bias.

Figure S1. Results of direct pairwise meta-analysis for weaning success

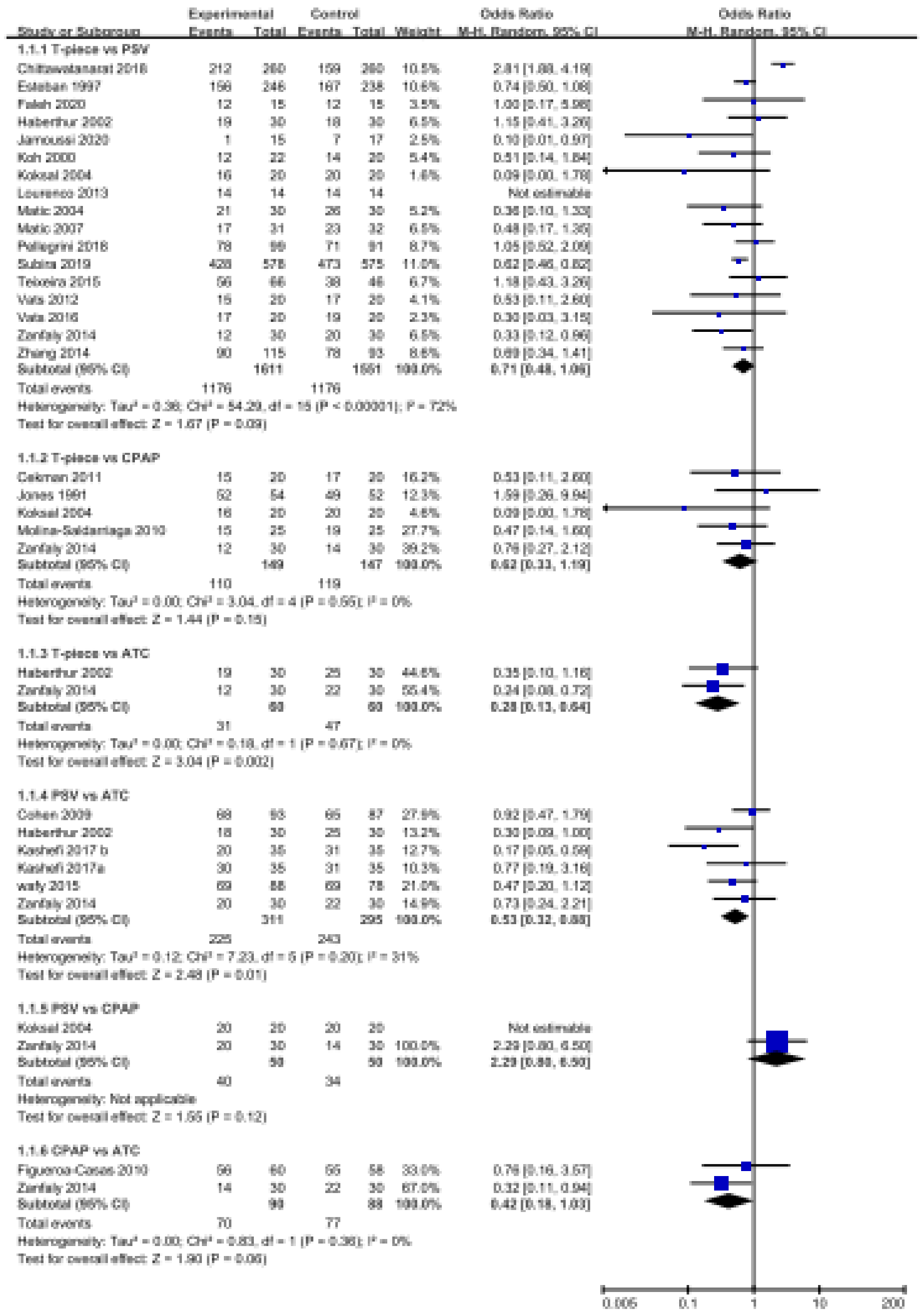

The summary effect estimate (odds ratio, OR) for individual randomized controlled trials is indicated by blue rectangles (the size of the rectangle is proportional to the study weight), with the black horizontal lines representing 95% confidence interval (CI). The overall summary effect estimate (OR) and 95% CI is indicated by the black diamond below.

Figure S2. Results of direct pairwise meta-analysis for reintubation

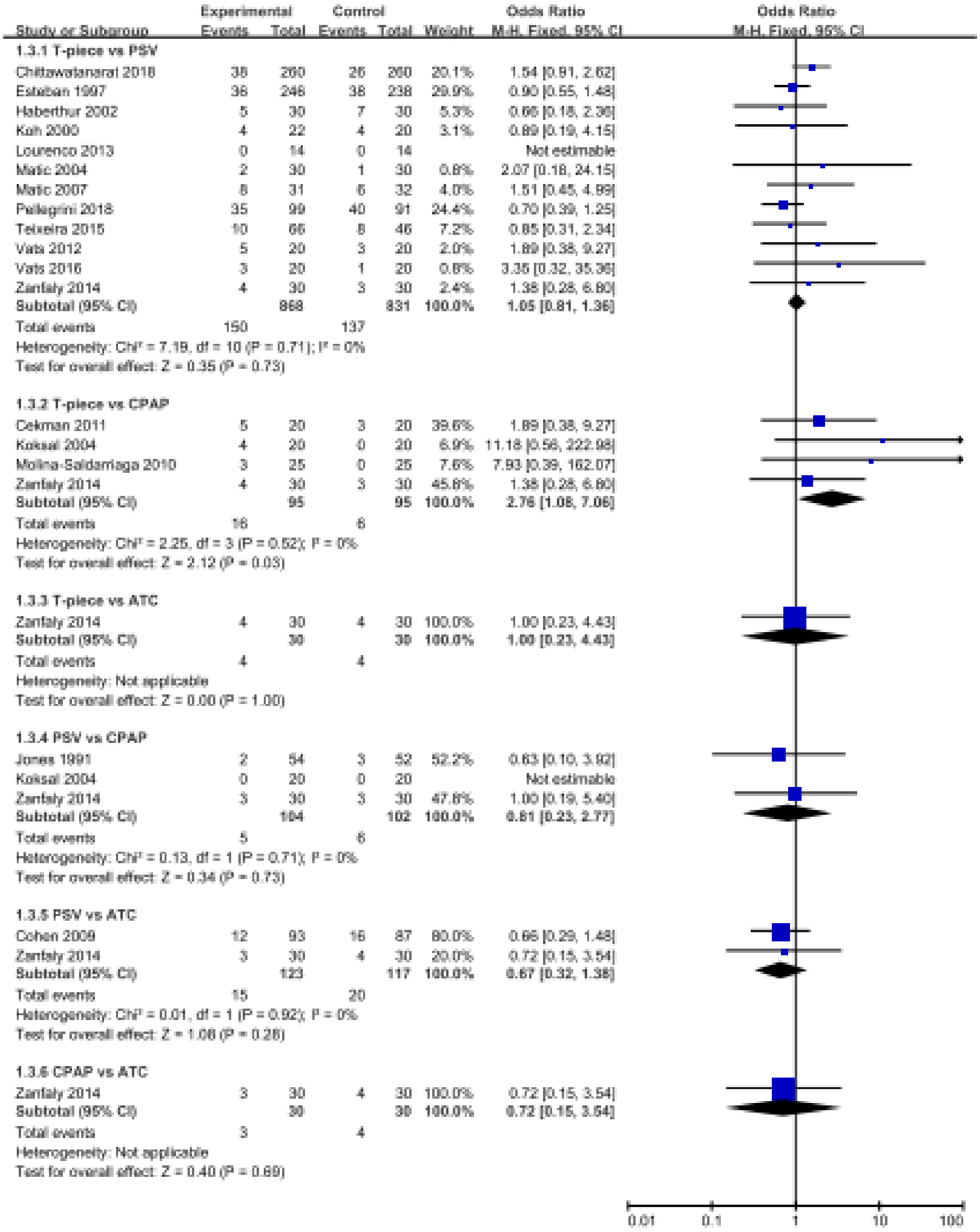

The summary effect estimate (odds ratio, OR) for individual randomized controlled trials is indicated by blue rectangles (the size of the rectangle is proportional to the study weight), with the black horizontal lines representing 95% confidence interval (CI). The overall summary effect estimate (OR) and 95% CI is indicated by the black diamond below.

Figure S3. Results of direct pairwise meta-analysis for SBT success

Study or Subgroup	Experimental Events	Experimental Total	Control Events	Control Total	Weight	Odds Ratio M-H, Fixed, 95% CI
**1.5.1 T-piece vs PSV**						
Esteban 1997	192	246	205	238	21.9%	0.57 [0.36, 0.92]
Haberthur 2002	24	30	25	30	2.4%	0.80 [0.22, 2.97]
Lourenco 2013	14	14	14	14		Not estimable
Matic 2004	80	110	120	150	13.3%	0.67 [0.37, 1.19]
Matic 2007	35	66	38	70	8.3%	0.95 [0.48, 1.87]
Subira 2019	486	578	532	575	40.7%	0.43 [0.29, 0.63]
Teixeira 2015	51	69	32	47	4.8%	1.33 [0.59, 3.00]
Vats 2012	15	20	17	20	2.0%	0.53 [0.11, 2.60]
Vats 2016	17	20	19	20	1.4%	0.30 [0.03, 3.15]
Zanfaly 2014	16	30	23	30	5.1%	0.36 [0.11, 1.05]
**Subtotal (95% CI)**		**1183**		**1194**	**100.0%**	**0.58 [0.47, 0.73]**
Total events	930		1025			
Heterogeneity: Chi² = 10.08, df = 8 (P = 0.26); I² = 21%						
Test for overall effect: Z = 4.78 (P < 0.00001)						
**1.5.2 T-piece vs CPAP**						
Molina-Saldarriaga 2010	18	25	19	25	40.1%	0.81 [0.23, 2.88]
Zanfaly 2014	16	30	17	30	59.9%	0.87 [0.32, 2.42]
**Subtotal (95% CI)**		**55**		**55**	**100.0%**	**0.85 [0.38, 1.88]**
Total events	34		36			
Heterogeneity: Chi² = 0.01, df = 1 (P = 0.93); I² = 0%						
Test for overall effect: Z = 0.40 (P = 0.69)						
**1.5.3 T-piece vs ATC**						
Haberthur 2002	24	30	29	30	32.3%	0.14 [0.02, 1.23]
Zanfaly 2014	16	30	26	30	67.7%	0.18 [0.05, 0.63]
**Subtotal (95% CI)**		**60**		**60**	**100.0%**	**0.16 [0.05, 0.49]**
Total events	40		55			
Heterogeneity: Chi² = 0.04, df = 1 (P = 0.85); I² = 0%						
Test for overall effect: Z = 3.22 (P = 0.001)						
**1.5.4 PSV vs CPAP**						
Zanfaly 2014	23	30	17	30	100.0%	2.51 [0.83, 7.64]
**Subtotal (95% CI)**		**30**		**30**	**100.0%**	**2.51 [0.83, 7.64]**
Total events	23		17			
Heterogeneity: Not applicable						
Test for overall effect: Z = 1.62 (P = 0.10)						
**1.5.5 PSV vs ATC**						
Cohen 2009	80	93	81	87	51.8%	0.46 [0.17, 1.26]
Haberthur 2002	25	30	29	30	21.4%	0.17 [0.02, 1.58]
Zanfaly 2014	23	30	26	30	26.8%	0.51 [0.13, 1.95]
**Subtotal (95% CI)**		**153**		**147**	**100.0%**	**0.41 [0.19, 0.87]**
Total events	128		136			
Heterogeneity: Chi² = 0.72, df = 2 (P = 0.70); I² = 0%						
Test for overall effect: Z = 2.33 (P = 0.02)						
**1.5.6 CPAP vs ATC**						
Figueroa-Casas 2010	52	60	56	58	40.3%	0.23 [0.05, 1.14]
Zanfaly 2014	17	30	26	30	59.7%	0.20 [0.06, 0.72]
**Subtotal (95% CI)**		**90**		**88**	**100.0%**	**0.21 [0.08, 0.58]**
Total events	69		82			
Heterogeneity: Chi² = 0.02, df = 1 (P = 0.89); I² = 0%						
Test for overall effect: Z = 3.03 (P = 0.002)						

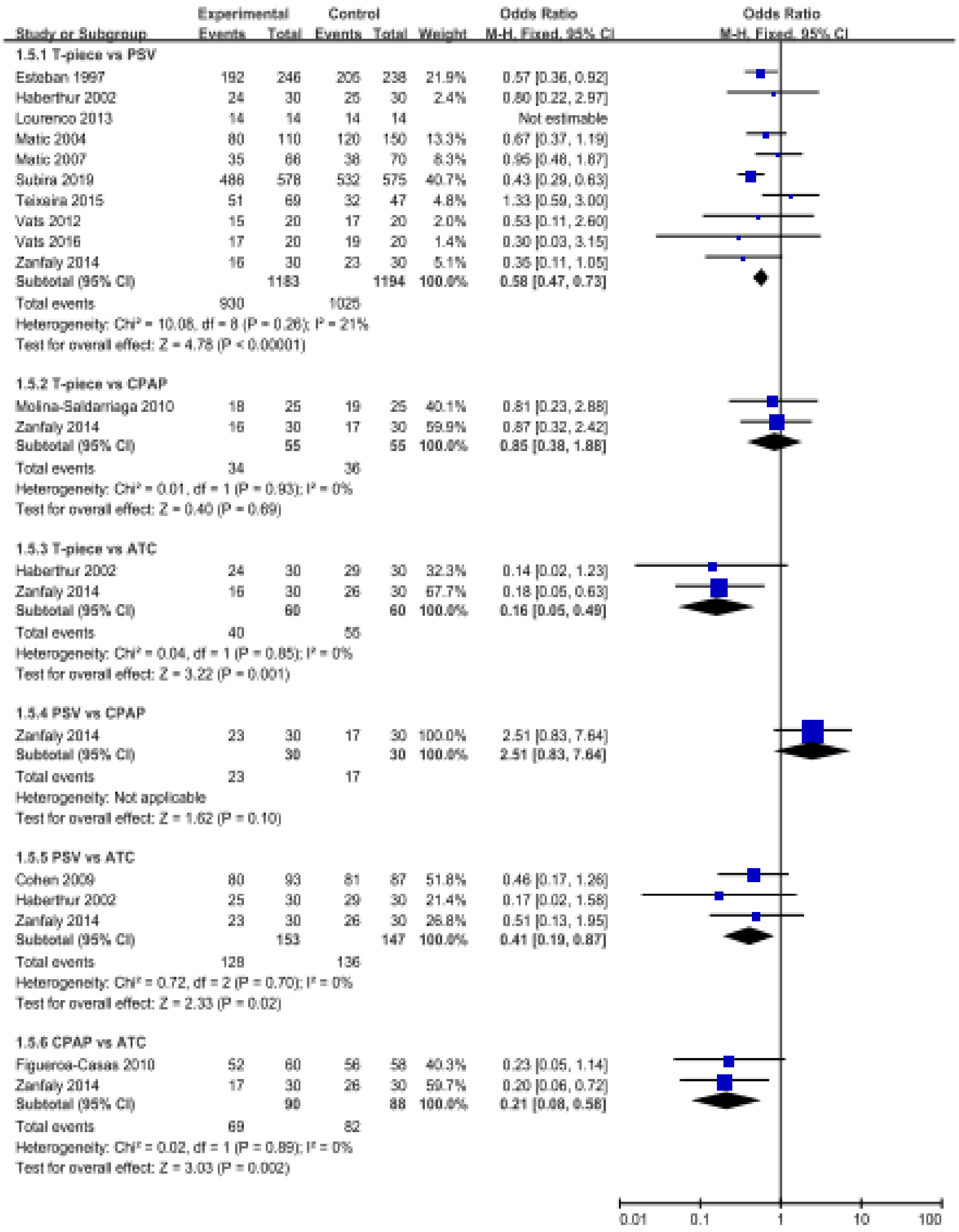

Test for subgroup differences: Chi² = 17.21, df = 5 (P = 0.004), I² = 70.9%

The summary effect estimate (odds ratio, OR) for individual randomized controlled trials is indicated by blue rectangles (the size of the rectangle is proportional to the study weight), with the black horizontal lines representing 95% confidence interval (CI). The overall summary effect estimate (OR) and 95% CI is indicated by the black diamond below.

Figure S4. Results of direct pairwise meta-analysis for ICU or LWU length of stay

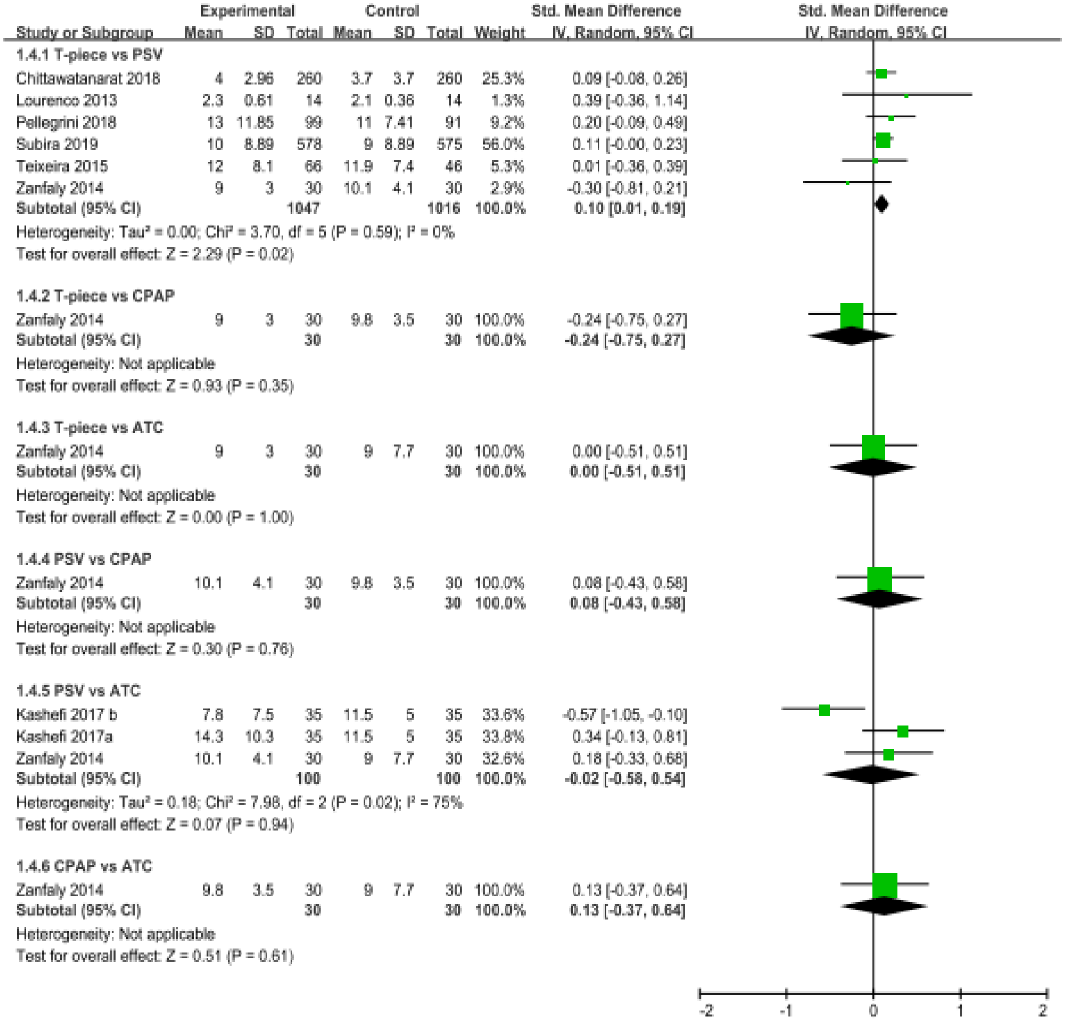

The summary effect estimate (odds ratio, OR) for individual randomized controlled trials is indicated by blue rectangles (the size of the rectangle is proportional to the study weight), with the black horizontal lines representing 95% confidence interval (CI). The overall summary effect estimate (OR) and 95% CI is indicated by the black diamond below.

Figure S5. Results of direct pairwise meta-analysis for ICU mortality (only for T-piece VS pressure support ventilation)

Study or Subgroup	Experimental Events	Experimental Total	Control Events	Control Total	Weight	Odds Ratio M-H, Random, 95% CI
Chittawatanarat 2018	9	260	8	260	8.9%	1.13 [0.43, 2.97]
Esteban 1997	28	246	21	238	23.5%	1.33 [0.73, 2.41]
Jamoussi 2020	2	15	7	17	2.7%	0.22 [0.04, 1.30]
Lourenco 2013	0	14	0	14		Not estimable
Matic 2004	4	30	2	30	2.6%	2.15 [0.36, 12.76]
Matic 2007	4	31	2	32	2.7%	2.22 [0.38, 13.11]
Pellegrini 2018	26	99	25	91	20.3%	0.94 [0.49, 1.79]
Subira 2019	38	578	29	575	33.8%	1.32 [0.81, 2.18]
Teixeira 2015	5	66	1	46	1.8%	3.69 [0.42, 32.67]
Vats 2012	4	20	5	20	3.8%	0.75 [0.17, 3.33]
**Total (95% CI)**		**1359**		**1323**	**100.0%**	**1.19 [0.89, 1.59]**
Total events	120		100			

Heterogeneity: Tau² = 0.00; Chi² = 6.62, df = 8 (P = 0.58); I² = 0%
Test for overall effect: Z = 1.17 (P = 0.24)

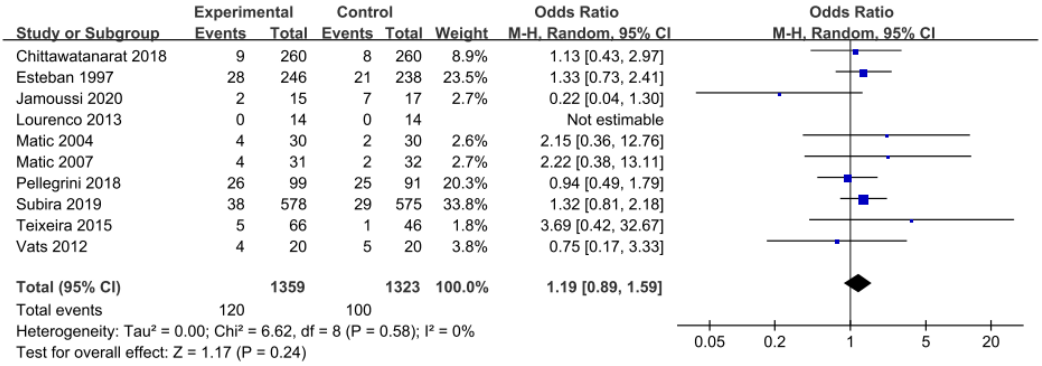

The summary effect estimate (odds ratio, OR) for individual randomized controlled trials is indicated by blue rectangles (the size of the rectangle is proportional to the study weight), with the black horizontal lines representing 95% confidence interval (CI). The overall summary effect estimate (OR) and 95% CI is indicated by the black diamond below.

Figure S6. Evaluation of inconsistency for four outcomes

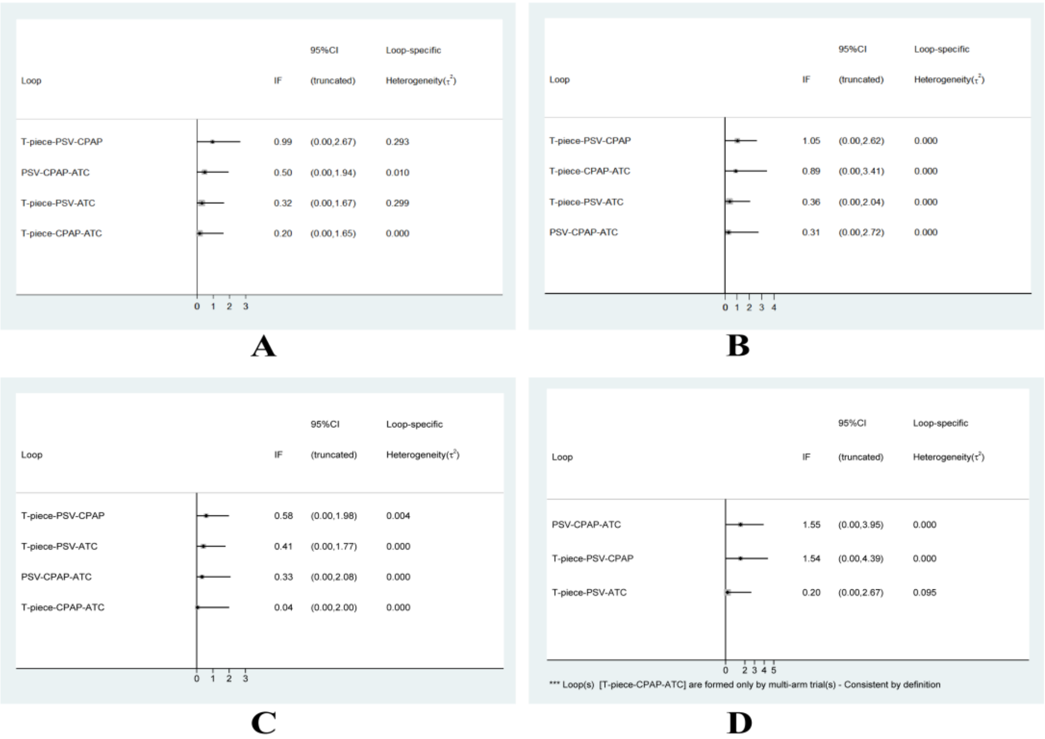

A, weaning success; B, reintubation; C, SBT success; and D, ICU or LWU length of stay. It indicates a consistency of evidences between direct and indirect comparisons if the lower limit of the 95% confidence intervals containing zero. PSV, pressure support ventilation; CPAP, continuous positive airway pressure; ATC, automatic tube compensation.

Figure S7. Predictive intervals plot for four outcomes

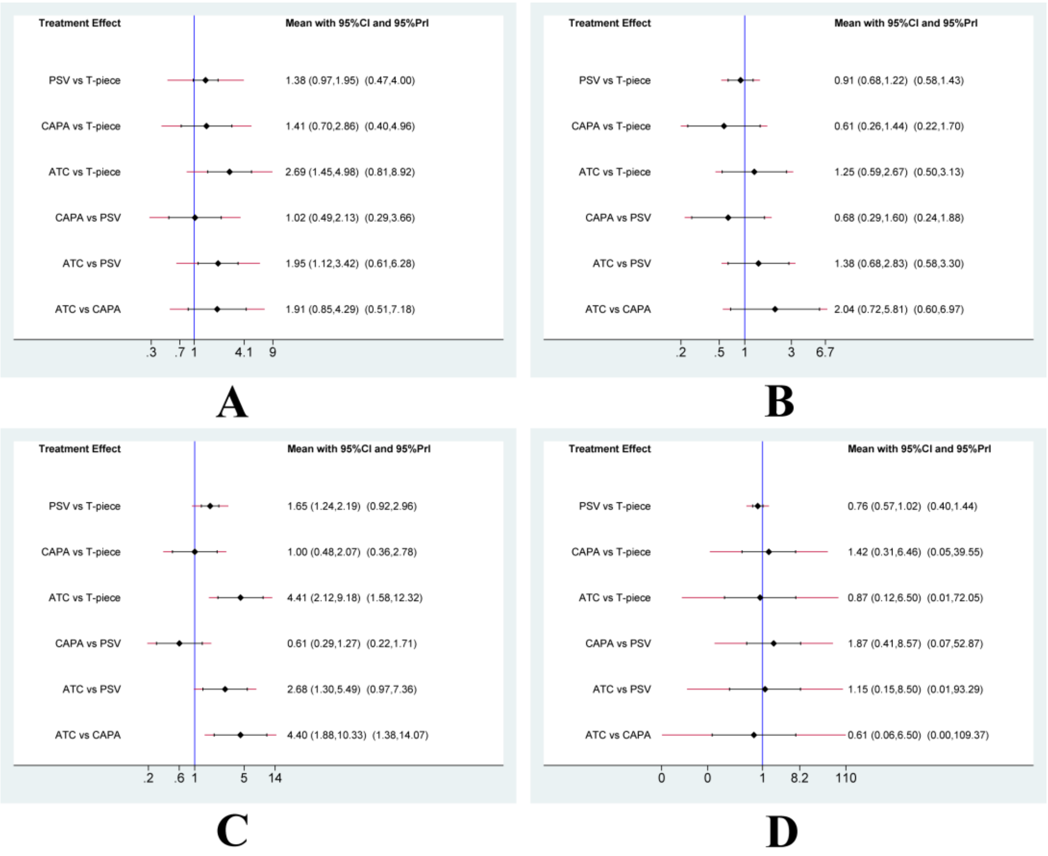

A, weaning success; B, reintubation; C, SBT success; and D, ICU or LWU length of stay. The graph presents the network estimates for all pairwise comparisons. Black horizontal lines represent the confidence intervals, and red horizontal lines represent the predictive intervals. The blue vertical line is the line of no effect. PSV, pressure support ventilation; CPAP, continuous positive airway pressure; ATC, automatic tube compensation.

Table S5. Ranking probabilities of different SBT technologies on four outcomes

Weaning technology	Weaning success		reintubation		SBT success		ICU or LWU length of stay	
	SUCRA	Rank	SUCRA	Rank	SUCRA	Rank	SUCRA	Rank
T-piece	0.852	4	0.517	2	0.498	3	0.443	2
PSV	0.527	3	0.592	3	0.891	2	0.423	3
CPAP	0.473	2	0.839	4	0.499	4	0.467	1
ATC	0.917	1	0.621	1	0.997	1	0.399	4

SUCRA, surface under the cumulative ranking curve; PSV, pressure support ventilation; CPAP, continuous positive airway pressure; ATC, automatic tube compensation.

Figure S8. Ranking of all SBT technologies

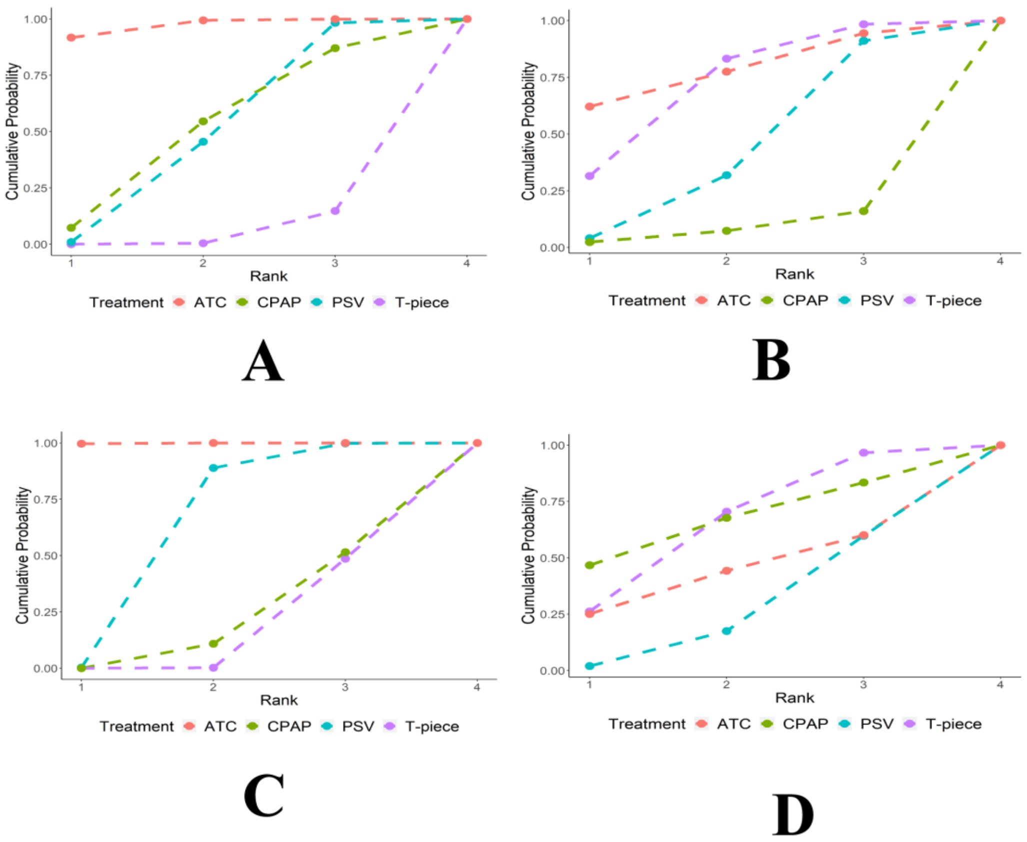

A, weaning success; B, reintubation; C, SBT success; and D, ICU or LWU length of stay. Larger SUCRA value suggests better results for the respective SBT technology. PSV, pressure support ventilation; CPAP, continuous positive airway pressure; ATC, automatic tube compensation; SUCRA, surface under the cumulative ranking curve.

Figure S9. Contribution plot for four outcomes

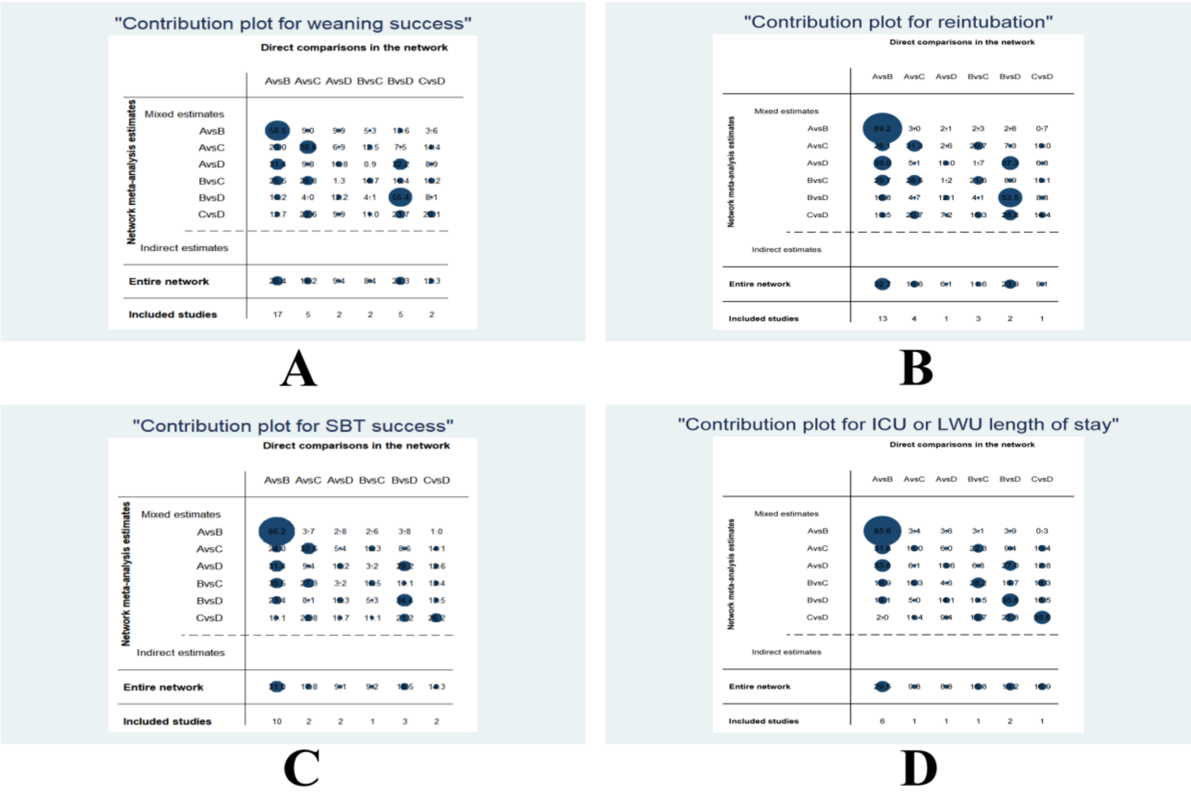

A, weaning success; B, reintubation; C, SBT success; and D, ICU or LWU length of stay. The size of the squares is proportional to the percentage contribution of the column-defining direct comparison to the row-defining network estimate. In the figure, A, B, C, and D indicates T-piece, PSV, CPAP, and ATC. PSV, pressure support ventilation; CPAP, continuous positive airway pressure; ATC, automatic tube compensation.

Figure S10. Comparison-adjusted funnel plot for four outcomes

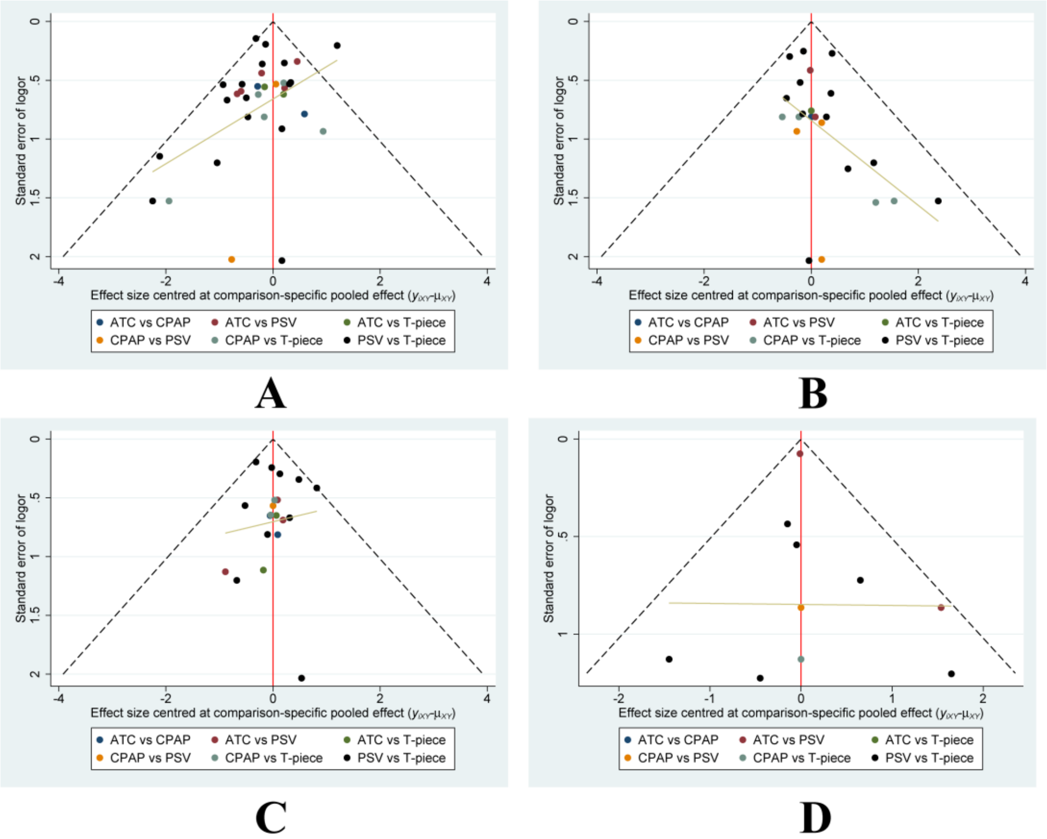

A, weaning success; B, reintubation; C, SBT success; and D, ICU or LWU length of stay. PSV, pressure support ventilation; CPAP, continuous positive airway pressure; ATC, automatic tube compensation.

Table S6. Evaluation of the quality of evidence for each outcome

Weaning success.

Comparison	Study limitation	Imprecision	Heterogeneity and inconsistency	Indirectness	Publication bias	Confidence in OR for weaning success
T-piece vs PSV	100% of the estimate from studies at moderate risk.	OR=0.68, 95%CI: 0.45 to 0.99	Moderate heterogeneity according to I^2(72%) and P-value (<0.001) in direct comparisons. No inconsistency between direct and indirect estimate (Node-split p=0.779).	no available information about effect modifiers	The funnel plot for the direct comparison is not suggestive of any dominant publication bias.	Low (Downgrade by two levels due to study limitation and indirectness)
T-piece vs CPAP	100% of the estimate from studies at moderate risk.	OR=0.66, 95%CI: 0.3 to 1.39	Mild heterogeneity according to I^2 (0%) and P-value (0.55) in direct comparisons. No inconsistency between direct and indirect estimate (Node-split p=0.996).	no available information about effect modifiers	Not enough to make funnel plots	Low (Downgrade by two levels due to study limitation and indirectness)
T-piece vs ATC	100% of the estimate from studies at moderate risk.	OR=0.34, 95%CI: 0.17 to 0.66	Mild heterogeneity according to I^2 (0%) and P-value (0.67) in direct comparisons.	no available information about effect modifiers	Not enough to make funnel plots	Low (Downgrade by two levels due to study

			No inconsistency between direct and indirect estimate (Node-split p=0.654).			limitation and indirectness)
PSV vs ATC	100% of the estimate from studies at moderate risk.	OR=0.5, 95%CI: 0.27 to 0.92	Mild heterogeneity according to I^2 (31%) and P-value (0.20) in direct comparisons. No inconsistency between direct and indirect estimate (Node-split p=0.244).	no available information about effect modifiers	Not enough to make funnel plots	Low (Downgrade by two levels due to study limitation and indirectness)
PSV vs CPAP	100% of the estimate from studies at moderate risk.	OR=0.96, 95%CI: 0.44 to 2.14	Only one head-to-head study can be estimable and no heterogeneity. No inconsistency between direct and indirect estimate (Node-split p=0.904).	no available information about effect modifiers	Not enough to make funnel plots	Low (Downgrade by two levels due to study limitation and indirectness)
CPAP vs ATC	100% of the estimate from studies at moderate risk.	OR=0.52, 95%CI: 0.21, 1.26	Mild heterogeneity according to I^2 (0%) and P-value (0.36) in direct comparisons. No inconsistency between direct and	no available information about effect modifiers	Not enough to make funnel plots	Low (Downgrade by two levels due to study limitation and indirectness)

			indirect estimate (Node-split p=0.623).			
Ranking of treatment	100% of the estimate from studies at moderate risk.	SUCRA plots suggested precision in a ranking of treatments.	No significant heterogeneity in network meta-analyses according to common τ^2 (0.22).<br><br>No significant inconsistency in test of global inconsistency (P = 0.6904), and few inconsistencies in local inconsistency.	no available information about effect modifiers	The comparison-adjusted funnel plot for the network is not suggestive of any dominant publication bias.	Low (Downgrade by two levels due to study limitation and indirectness)

PSV, pressure support ventilation; CPAP, continuous positive airway pressure; ATC, automatic tube compensation.

ICU mortality.

Comparison	Study limitation	Imprecision	Heterogeneity and inconsistency	Indirectness	Publication bias	Confidence in OR for weaning success
T-piece vs PSV	100% of the estimate from studies at low risk.	OR=0.68, 95%CI: 0.45 to 0.99	Mild heterogeneity according to I^2(0%) and P-value (0.58) in direct comparisons.	no available information about effect modifiers	The funnel plot for the direct comparison is not suggestive of any dominant publication bias.	Moderate (Downgrade by one level due to indirectness)

PSV, pressure support ventilation; CPAP, continuous positive airway pressure; ATC, automatic tube compensation.

Reintubation.

Comparison	Study limitation	Imprecision	Heterogeneity and inconsistency	Indirectness	Publication bias	Confidence in OR for weaning success
T-piece vs PSV	100% of the estimate from studies at moderate risk.	OR=1.15, 95%CI: 0.82 to 1.84	Mild heterogeneity according to I^2(0%) and P-value(0.71) in direct comparisons. No inconsistency between direct and indirect estimate (Node-split p=0.189).	no available information about effect modifiers	The funnel plot for the direct comparison is not suggestive of any dominant publication bias.	Low (Downgrade by two levels due to study limitation and indirectness)
T-piece vs CPAP	100% of the estimate from studies at moderate risk.	OR=2, 95%CI: 0.83 to 5.48)	Mild heterogeneity according to I^2 (0%) and P-value (0.52) in direct comparisons. No inconsistency between direct and indirect estimate (Node-split p=0.135).	no available information about effect modifiers	Not enough to make funnel plots	Low (Downgrade by two levels due to study limitation and indirectness)
T-piece vs ATC	100% of the estimate from studies at moderate risk.	OR=0.85, 95%CI: 0.35 to 2.3	Only one head-to-head study can be estimable and no heterogeneity. No inconsistency	no available information about effect modifiers	Not enough to make funnel plots	Low (Downgrade by two levels due to study

			between direct and indirect estimate (Node-split p=0.794).			limitation and indirectness)
PSV vs ATC	100% of the estimate from studies at moderate risk.	OR=0.5, 95%CI: 0.27 to 0.92	Mild heterogeneity according to I^2 (0%) and P-value (0.92) in direct comparisons.	no available information about effect modifiers	Not enough to make funnel plots	Low (Downgrade by two levels due to study limitation and indirectness)
PSV vs CPAP	100% of the estimate from studies at moderate risk.	OR=1.71, 95%CI: 0.7 to 4.62	Mild heterogeneity according to I^2 (0%) and P-value (0.71) in direct comparisons. No inconsistency between direct and indirect estimate (Node-split p=0.151).	no available information about effect modifiers	Not enough to make funnel plots	Low (Downgrade by two levels due to study limitation and indirectness)
CPAP vs ATC	100% of the estimate from studies at moderate risk.	OR=0.42, 95%CI: 0.13 to 1.38	Only one head-to-head study can be estimable and no heterogeneity. No inconsistency between direct and indirect estimate (Node-split p=0.478).	no available information about effect modifiers	Not enough to make funnel plots	Low (Downgrade by two levels due to study limitation and indirectness)

Ranking of treatment	100% of the estimate from studies at moderate risk.	SUCRA plots suggested precision in a ranking of treatments.	No significant heterogeneity in network meta-analyses according to common τ^2 (0.02).<br><br>No significant inconsistency in test of global inconsistency (P = 0.5229), and few inconsistencies in local inconsistency.	no available information about effect modifiers	The comparison-adjusted funnel plot for the network is not suggestive of any dominant publication bias.	Low (Downgrade by two levels due to study limitation and indirectness)

PSV, pressure support ventilation; CPAP, continuous positive airway pressure; ATC, automatic tube compensation.

ICU or LWU length of stay.

Comparison	Study limitation	Imprecision	Heterogeneity and inconsistency	Indirectness	Publication bias	Confidence in OR for weaning success
T-piece vs PSV	85.6% of the estimate from studies at low risk and 14.4% at moderate risk.	OR=1.52, 95%CI: 0.74, to 6.91	Mild heterogeneity according to I^2(0%) and P-value (0.59) in direct comparisons.	no available information about effect modifiers	The funnel plot for the direct comparison is not suggestive of any dominant publication bias.	Moderate (Downgrade by two levels due to study limitation and indirectness)
T-piece vs CPAP	31.8% of the estimate from studies at low risk and 68.2% at moderate risk.	OR=0.82, 95%CI: 0.08 to 11.66)	Only one head-to-head study can be estimable and no heterogeneity.	no available information about effect modifiers	Not enough to make funnel plots	Low (Downgrade by two levels due to study limitation and indirectness)
T-piece vs ATC	33.8% of the estimate from studies at low risk and 66.2% at moderate risk.	OR=1.34, 95%CI: 0.12 to 20.86	Only one head-to-head study can be estimable and no heterogeneity. No inconsistency between direct and indirect estimate (Node-split p=0.905).	no available information about effect modifiers	Not enough to make funnel plots	Low (Downgrade by two levels due to study limitation and indirectness)
PSV vs ATC	19.1% of the	OR=0.84, 95%CI:	Moderate heterogeneity	no available	Not enough to make	Low

	estimate from studies at low risk and 80.9% at moderate risk.	0.07 to 9.67	according to I^2 (75%) and P-value (0.02) in direct comparisons.	information about effect modifiers	funnel plots	(Downgrade by two levels due to study limitation and indirectness)
PSV vs CPAP	19.9% of the estimate from studies at low risk and 80.1% at moderate risk	OR=0.52, 95%CI: 0.04 to 5.23	Only one head-to-head study can be estimable and no heterogeneity.	no available information about effect modifiers	Not enough to make funnel plots	Low (Downgrade by two levels due to study limitation and indirectness)
CPAP vs ATC	2% of the estimate from studies at low risk and 98% at moderate risk	OR=1.64, 95%CI: 0.08, to 40.63	Only one head-to-head study can be estimable and no heterogeneity.	no available information about effect modifiers	Not enough to make funnel plots	Low (Downgrade by two levels due to study limitation and indirectness)
Ranking of treatment	100% at moderate risk	SUCRA plots suggested precision in a ranking of treatments.	No significant heterogeneity in network meta-analyses according to common τ^2 (<0.000).	no available information about effect modifiers	The comparison-adjusted funnel plot for the network is not suggestive of any dominant publication bias.	Low (Downgrade by two levels due to study limitation and indirectness)

			No significant inconsistency in test of global inconsistency (P = 0.3084), and few inconsistencies in local inconsistency.			

PSV, pressure support ventilation; CPAP, continuous positive airway pressure; ATC, automatic tube compensation.

SBT success.

Comparison	Study limitation	Imprecision	Heterogeneity and inconsistency	Indirectness	Publication bias	Confidence in OR for weaning success
T-piece vs PSV	100% of the estimate from studies at moderate risk.	OR=1.15, 95%CI: 0.82 to 1.84	Mild heterogeneity according to I^2(21%) and P-value (0.26) in direct comparisons. No significant inconsistency in test of global inconsistency (P = 0.653), and few inconsistencies in local inconsistency.	no available information about effect modifiers	The funnel plot for the direct comparison is not suggestive of any dominant publication bias.	Low (Downgrade by two levels due to study limitation and indirectness)
T-piece vs CPAP	100% of the estimate from studies at moderate risk.	OR=2, 95%CI: 0.83 to 5.48)	Mild heterogeneity according to I^2(0%) and P-value (0.93) in direct comparisons. No significant inconsistency in test of global inconsistency (P = 0.757), and few inconsistencies in local inconsistency.	no available information about effect modifiers	Not enough to make funnel plots	Low (Downgrade by two levels due to study limitation and indirectness)
T-piece vs ATC	100% of the	OR=0.85, 95%CI:	Mild heterogeneity	no available	Not enough to make	Low

	estimate from studies at moderate risk.	0.35 to 2.3	according to I^2(0%) and P-value (0.85) in direct comparisons. No inconsistency between direct and indirect estimate (Node-split p=0.497).	information about effect modifiers	funnel plots	(Downgrade by two levels due to study limitation and indirectness)
PSV vs ATC	100% of the estimate from studies at moderate risk.	OR=0.5, 95%CI: 0.27 to 0.92	Mild heterogeneity according to I^2 (0%) and P-value (0.70) in direct comparisons. No inconsistency between direct and indirect estimate (Node-split p=0.755).	no available information about effect modifiers	Not enough to make funnel plots	Low (Downgrade by two levels due to study limitation and indirectness)
PSV vs CPAP	100% of the estimate from studies at moderate risk.	OR=1.71, 95%CI: 0.7 to 4.62	Only one head-to-head study can be estimable and no heterogeneity. No inconsistency between direct and indirect estimate (Node-split p=0.471).	no available information about effect modifiers	Not enough to make funnel plots	Low (Downgrade by two levels due to study limitation and indirectness)
CPAP vs ATC	100% of the estimate from studies at moderate risk.	OR=0.42, 95%CI: 0.13 to 1.38	Mild heterogeneity according to I^2 (0%) and P-value (0.89) in direct comparisons.	no available information about effect modifiers	Not enough to make funnel plots	Low (Downgrade by two levels due to study

			No inconsistency between direct and indirect estimate (Node-split p=0.809).			limitation and indirectness)
Ranking of treatment	100% of the estimate from studies at moderate risk.	SUCRA plots suggested precision in a ranking of treatments.	No significant heterogeneity in network meta-analyses according to common τ^2 (0.04).<br><br>No significant inconsistency in test of global inconsistency (P = 0.9513), and few inconsistencies in local inconsistency.	no available information about effect modifiers	The comparison-adjusted funnel plot for the network is not suggestive of any dominant publication bias.	Low (Downgrade by two levels due to study limitation and indirectness)

PSV, pressure support ventilation; CPAP, continuous positive airway pressure; ATC, automatic tube compensation; SBT, spontaneous breathing trial.
